# Epigenetic Characterization of the Growth Hormone Gene Identifies SmcHD1 as a Regulator of Autosomal Gene Clusters

**DOI:** 10.1371/journal.pone.0097535

**Published:** 2014-05-12

**Authors:** Shabnam Massah, Robert Hollebakken, Mark P. Labrecque, Addie M. Kolybaba, Timothy V. Beischlag, Gratien G. Prefontaine

**Affiliations:** 1 Faculty of Health Sciences, Simon Fraser University, Burnaby, BC, Canada; 2 Faculty of Biology, Ludwig Maximilians University Munich, Martinsried, Germany; CNRS, France

## Abstract

Regulatory elements for the mouse growth hormone (GH) gene are located distally in a putative locus control region (LCR) in addition to key elements in the promoter proximal region. The role of promoter DNA methylation for GH gene regulation is not well understood. Pit-1 is a POU transcription factor required for normal pituitary development and obligatory for GH gene expression. In mammals, Pit-1 mutations eliminate GH production resulting in a dwarf phenotype. In this study, dwarf mice illustrated that Pit-1 function was obligatory for GH promoter hypomethylation. By monitoring promoter methylation levels during developmental GH expression we found that the GH promoter became hypomethylated coincident with gene expression. We identified a promoter *differentially methylated region* (DMR) that was used to characterize a methylation-dependent DNA binding activity. Upon DNA affinity purification using the DMR and nuclear extracts, we identified structural maintenance of chromosomes hinge domain containing -1 (SmcHD1). To better understand the role of SmcHD1 in genome-wide gene expression, we performed microarray analysis and compared changes in gene expression upon reduced levels of SmcHD1 in human cells. Knock-down of SmcHD1 in human embryonic kidney (HEK293) cells revealed a disproportionate number of up-regulated genes were located on the X-chromosome, but also suggested regulation of genes on non-sex chromosomes. Among those, we identified several genes located in the protocadherin β cluster. In addition, we found that imprinted genes in the H19/Igf2 cluster associated with Beckwith-Wiedemann and Silver-Russell syndromes (BWS & SRS) were dysregulated. For the first time using human cells, we showed that SmcHD1 is an important regulator of imprinted and clustered genes.

## Introduction

Plasma growth hormone (GH) levels decline with age and contribute to decreased somatotropic axis signaling (GH releasing hormone [GHRH], GH and insulin-like growth factor -1 [IGF-1]) [1]. Understanding how GH is regulated will provide insight into events associated with declining levels of GH with age. It has been proposed that increasing GH levels in the elderly increases lean muscle tissue while decreasing adipose mass and may act to reverse some negative effects associated with aging [2]. The mammalian GH gene is expressed only in the pituitary and is dependent on the expression of a functional homeodomain containing transcription factor, the pituitary-specific Pit-1 protein (POU1-F1) [3]. During development in the absence of a functional Pit-1 protein, GH is not expressed resulting in a dwarf phenotype in mammals.

A distal locus control region (LCR) located ∼14.5 kb upstream of the human GH-N gene is required for gene expression [4]. It is characterized by a series of pituitary-specific DNase I hypersensitive sites (HS) when expressed. The region representing the homologous LCR in rodent models is relatively uncharacterized, while the human LCR encompassing HSI and HSII represents an intergenic sequence that is homologous to mouse and rat genomic sequence.

The GH promoter is regulated by both positive and negative DNA elements through transcription factors and co-regulatory proteins [5]. Pit-1 binds to DNA elements in the promoter as well as the LCR [6]. In addition, promoter DNA methylation has been negatively correlated with gene transcription; loss of DNA methylation near the transcriptional start site is associated with GH gene expression [7]–[9].

Distally located DNA elements communicate with the promoter to regulate gene expression. Recombined bacterial artificial chromosome (BAC) transgenes in which distal elements are deleted, have proved useful for studying the influence of these elements on DNA methylation in cis [10]. Here we report the characterization of GH promoter DNA methylation in which the putative mouse LCR was deleted. The goal was to impair transcriptional expression via removal of the putative LCR to determine its influence on promoter CpGs methylation. We hypothesized that the hypermethylation would denote CpGs crucial for gene repression while the same CpGs when hypomethylated would be essential for gene expression. We defined this region as a differentially methylated region (DMR). The direct role of promoter DNA methylation in regulation of the GH gene is not understood. Factors responsible for mediating DNA methylation dependent repression of the GH gene have not been identified. The ultimate goal was to identify proteins that bind to the methylated GH DMR and promote transcriptional repression. These studies should shed light on molecular mechanisms directing long-term repression of the GH gene.

Our findings indicate that Structural Maintenance of Chromosomes hinge domain containing-1 (SmcHD1) is a protein that can interact with the GH promoter and likely regulates its expression. SmcHD1 is a non-canonical member of the structural maintenance of chromosome (Smc) family. This family of proteins plays roles in chromatin dynamics and condensation and DNA repair [11], [12]. These roles establish DNA topology linking chromatin architecture with gene regulatory events. The Smc family of proteins is characterized by a conserved ATPase globular domain consisting of N- and C- terminal Walker A and B motifs characteristic of ABC-transporter ATPases [11]. SmcHD1 lacks these discernable ATPase motifs found in authentic Smc proteins and instead the ATPase domain of SmcHD1 resembles a GHKL (gyrase, HSP90, histidine kinase, MutL) ATPase domain found in the ATPase/kinase superfamily [13]. SmcHD1 homologues are found in vertebrates and in some plants [14].

The first part of this study focuses on the identification of a DMR, characterization of the binding of a DNA methylation dependent protein and isolation of proteins that bind to the DMR. For the first time, we show that SmcHD1 is likely a GH regulatory protein acting directly through the promoter. In the second part, we used a genome wide approach to identify autosomal genes regulated by SmcHD1. We confirm that SmcHD1 can repress genes in the protocadherin β gene cluster and extends its targets to a gene cluster with parent-of-origin imprinting associated with Beckwith-Wiedemann/Silver-Russell syndromes (BWS/SRS, respectively).

## Results

### Identification of a GH promoter differential methylated region (DMR)

**Figure 1:**
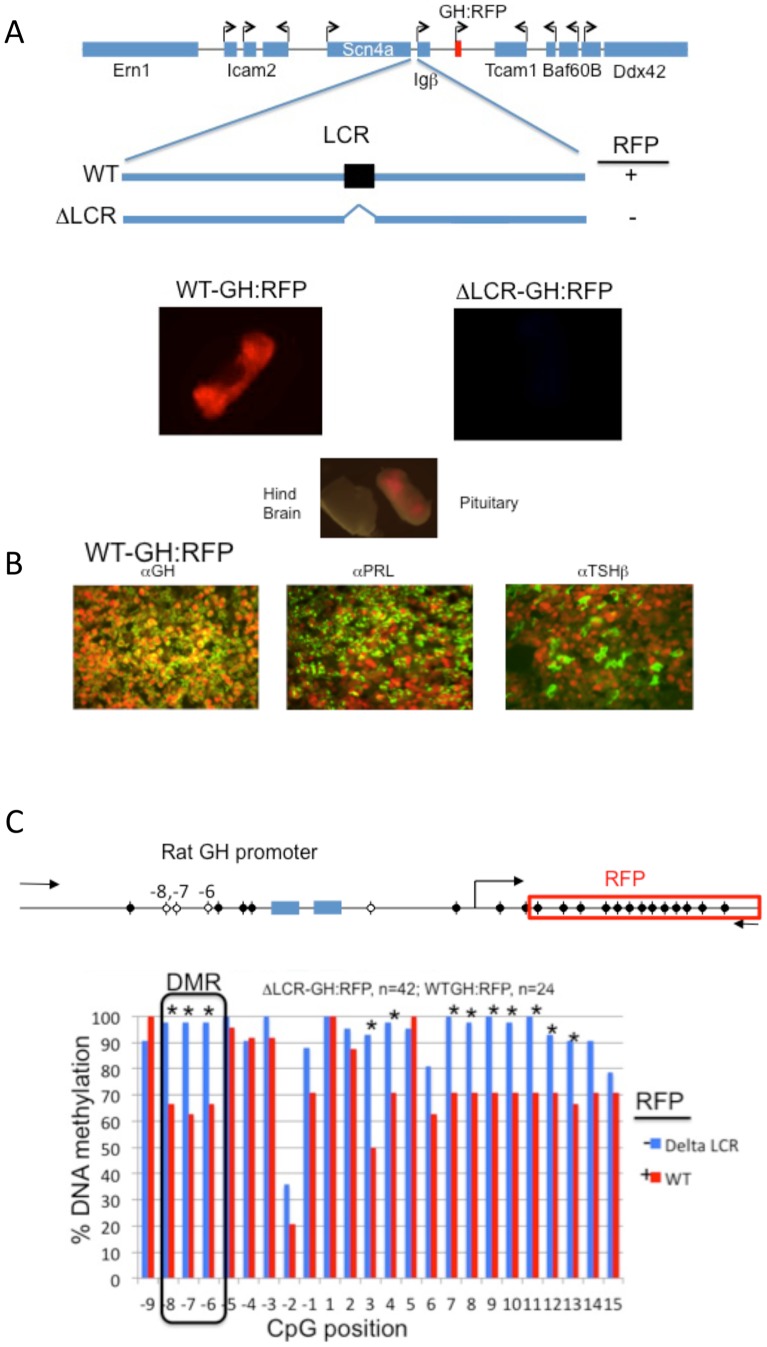
Identification of a GH promoter DMR using mouse BAC transgenes. **A.** A mouse BAC transgene encompassing GH gene was modified by homologous recombination to replace the mouse GH gene with the rat GH proximal promoter and the coding sequence for RFP. A second recombination event using the same BAC transgene was used to delete a putative distal LCR (ΔLCR- GH:RFP). Imaging of dissected mouse pituitary, illustrates RFP expression from the WT-GH:RFP but not ΔLCR-GH:RFP transgenic mice. **B.** WT-GH:RFP protein mirrored endogenous GH expression. Pituitaries were fixed and embedded in paraffin, the tissue sectioned and subjected to indirect immunofluorescence with anti-GH, anti-Prl and anti-TSHβ antibodies using Cy2-cojugated secondary antibodies. The images were merged with images of RFP expression. Note: RFP was nuclear while the hormones were localized to the cytoplasm. **C.** Pairwise analysis of the rat GH promoter methylation levels by bisulfite sequencing of pituitary DNA from WT-GH:RFP and ΔLCR-GH:RFP transgenic mice. The number indicates the relative position of the CpG from the transcriptional start site. The n-values indicate the number of clones sequenced from transgenic mice and used for the pairwise statistical analysis.

Several reports have identified DNA methylation as a regulatory component for expression of the pituitary-specific GH gene [7]–[9]. We found that genomic DNA from mouse pituitary was hypomethylated over the GH promoter region. In order to study GH gene expression in real-time using mouse pituitary, we generated a series of BAC transgenic mice where the mouse GH gene was substituted with a homologous rat GH promoter and the coding sequence for red fluorescent reporter gene (wild-type, WT-GH:RFP) and another that contained an additional deletion in a putative upstream regulatory element or locus control region (LCR, ΔLCR-GH:RFP). The mouse LCR was identified by homology with the human locus [6], [15]. Transgenic mice carrying the recombined WT-GH:RFP BAC but not the ΔLCR-GH:RFP BAC expressed RFP only in the pituitary (Figure 1A).

Normally GH is expressed in somatotropes (GH+) and in some somatomamotropes (GH+ and prolactin, Prl+). To show that the RFP protein expression closely paralleled GH expression using the WT BAC, we labeled pituitary tissue sections from mice with anti-GH, anti-Prl and anti- thyroid stimulating hormone β (TSHβ) antibodies and visualized the presence of each cytoplasmic hormone with a fluorescein isothiocyanate (FITC)-conjugated secondary antibody (green, Figure 1B). These results demonstrated that pituitary of transgenic mice containing WT BAC transgenes is a suitable model system to study promoter DNA methylation levels compared to the ΔLCR version of the BAC transgene, which lacked RFP expression.

Next, we assessed the level of DNA methylation on the proximal promoter of the mouse transgenes. Genomic DNA was isolated from pituitaries and sequenced following bisulfite treatment. A number of clones were analyzed using pairwise statistics (displayed in a graphic plot in Figure 1C). Expression of GH:RFP paralleling GH expression coincided with significantly hypomethylated CpGs at positions −8 through −6 as well as some in the RFP coding region. These same CpGs were fully methylated in pituitary tissues from the ΔLCR transgenic mice where GH:RFP was silenced. In addition, pyrosequencing the targeted promoter CpGs demonstrated a similar trend (Figure S1). In conclusion, GH:RFP BAC transgenic mice expressing RFP had significantly hypomethylated CpGs at position −8 through −6 of the promoter region compared to transgenic mice, ΔLCR GH:RFP.

### The GH promoter is heavily DNA methylated in dwarf mice pituitaries

**Figure 2:**
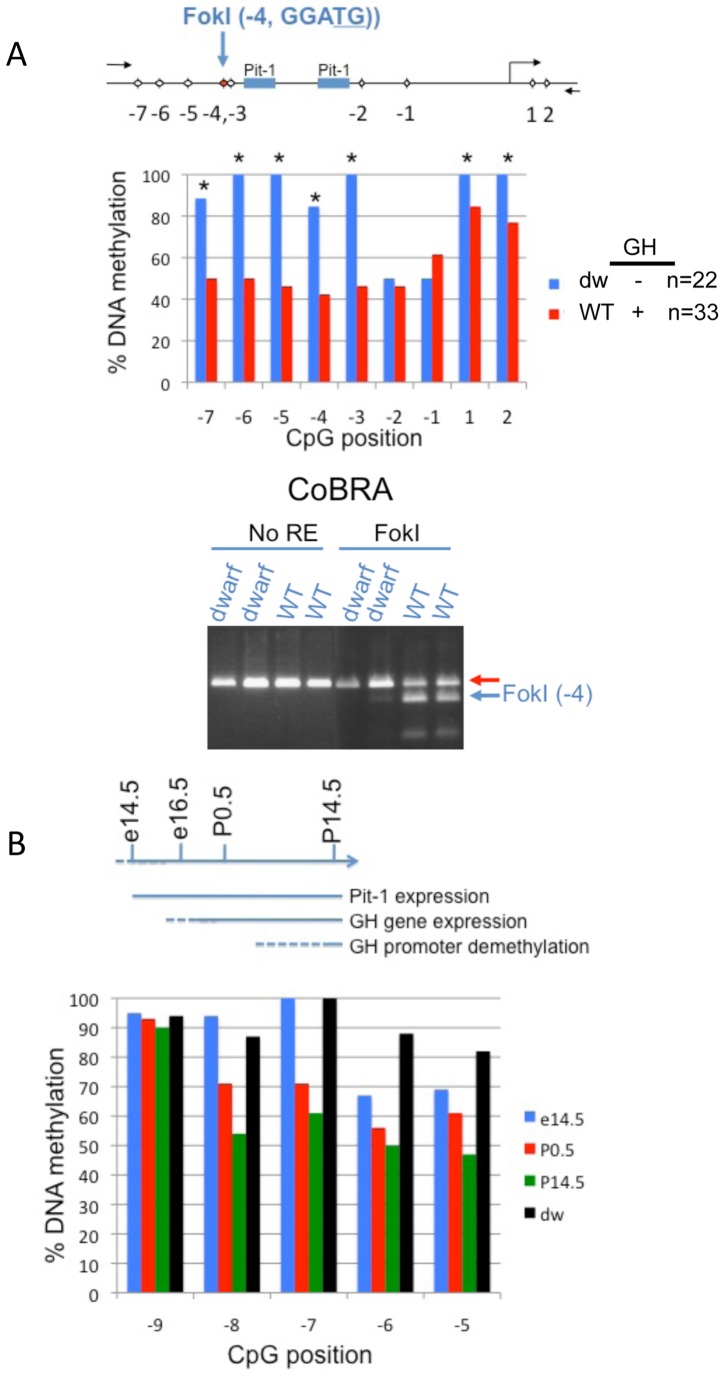
Comparative levels of GH promoter methylation in mice. **A.** Above, schematic of the relative position of CpGs in the mouse GH promoter. Middle, pairwise analysis comparing the levels of DNA methylation in wild type (WT) with dwarf (dw) mice (Snell) using bisulfite DNA sequencing. Bottom, combined bisulfite restriction analysis (CoBRA) of the CpG located at position −4 on the same samples. The proportion of non-methylated C nucleotide is indicated by the cleaved FokI products (blue arrow). **B.** The GH promoter becomes demethylated during mouse development and is coincident with GH gene expression. Above, schematic illustration of the developmental events relating to Pit-1-mediated induction of the GH gene and concomitant loss of GH promoter methylation. Below, pyrosequencing of bisulfite-treated genomic DNA extracted from mouse pituitary, selected from different days of mouse development (e14.5, P0.5 or P14.5) and displayed as the percent methylation of CpG sites in the mouse promoter.

Functional Pit-1 protein is required for expression of anterior pituitary secreted hormones including GH [3], [16]. In order to assess the level of promoter DNA methylation in pituitary cells lacking anterior pituitary hormones (GH, Prl and TSHβ), we compared DNA methylation levels in pituitaries from Snell dwarf mice (Dw) with heterozygous wild type littermates (WT). Dw mice are characterized by an inactivating Pit-1 mutation [17]. To determine the level of methylation, we quantified bisulfite treated DNA using two methods. First, we directly sequenced a number of clones produced from PCR reactions following bisulfite treatment of genomic DNA (Figure 2A, upper panel). The GH promoter from dwarf mouse pituitaries was almost completely methylated immediately upstream of the Pit-1 binding sites (Figure 2A, CpGs −7 through −3, solid blue bars). Conversely, WT samples were significantly hypomethylated DNA at CpG positions −7 through −3, consistent with transgenic mice carrying the rat GH promoter (Figure 2A, solid red bars). In a second experiment, following bisulfite treatment of the genomic DNA, the subsequent PCR product was interrogated by DNA restriction digest targeting the CpG located at position −4 and showed that DNA hypomethylation was only observed in the anterior pituitary from WT mice (WT, Figure 2A, lower panel). Together, these results demonstrate that the hypomethylated promoter of the mouse GH gene was attributed to cells with functional Pit-1.

### Postnatal hypomethylation of the pituitary GH promoter

The developmental profile of GH gene expression in mice is well established [17]. GH gene expression can be detected at around embryonic day (e) 15.5–e17.5 but not at e14.5. We were determined to characterize whether promoter demethylation correlated with the developmental expression of the GH gene: e14.5 (before GH expression), P0 (after GH expression) and P14.5 (postnatal GH expression). Bisulfite treated DNA and pyrosequencing revealed promoter hypomethylation following GH gene expression including CpGs −8 to −5 (Figure 2B). Therefore the GH promoter becomes hypomethylated at a developmental time shortly after GH gene expression.

### Treatment of cells with 5-azaC relieves GH transcriptional repression

**Figure 3:**
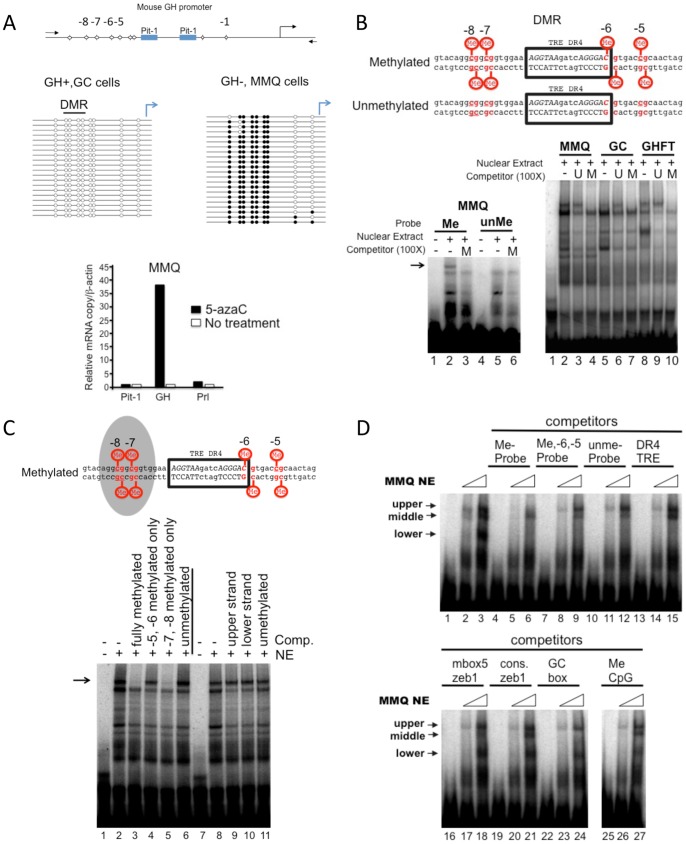
Inhibition of DNA methylation relieves transcriptional repression of GH and characterization of a methyl-DNA binding activity. **A.** GH gene repression can be relieved by treatment with 5-azaC. Above, schematic illustration of the CpG sites location in the rat GH promoter (the CpGs are numbered according to their relative position from the transcriptional start site). Middle, bisulfite sequencing of genomic DNA extracted from pituitary derived-GH+ (GC cells) or GH− cells (MMQ cells). The level of DNA methylation is displayed as unmethylated (open circles) or methylated (solid circles) from individual clones. Lower, GH− cells (MMQ) were treated with 5-azaC and the level of gene expression compared using RT-qPCR. The solid bars represent 5-azaC treated cells and the open bars, cells cultured under normal conditions. **B.** A methyl-specific binding protein binds to a region of DNA derived from the mouse GH promoter. Above, DNA sequence of the double-stranded oligonucleotides used in the EMSA. The position of the CpGs relative to the transcriptional start site are indicated and the position of a previously described binding site for the thyroid hormone receptor (TR) is boxed. Lower left, EMSA with MMQ nuclear extracts comparing the bound proteins from either methylated (lane 2) or unmethylated DNA probes (lane 5). A methylated competitor DNA (M) reveals a specific upper methyl-DNA protein-binding component (Lane 3, arrow). Lower right, an EMSA competition assay with nuclear extracts from pituitary-derived cell lines (MMQ, GC and GHFT) as indicated. Methylated or unmethylated (U) competitor oligonucleotides were used to identify the upper shifted component representing a DNA bound protein. All nuclear extracts contained a methyl-DNA binding protein. **C.** Methylation of CpGs at position −8 and −7 were required for recruiting a methyl DNA binding protein. Above, schematic of the result from the competition assay illustrated below. The shaded area indicates CpGs essential for the methyl DNA binding protein. Below, an EMSA with MMQ nuclear extracts (NE) and a methylated DNA probe with various competitor oligonucleotides as indicated. **D.** The methyl-DNA binding activity observed with the GH DMR appears to be unique. The sequence of the oligonucleotides used in the competition assay are listed in Table S1. An EMSA with increasing amounts of MMQ NE (6 and 12 µg) and a methylated GH DMR probe optionally in the presence of competitor oligonucleotides (100X) as indicated.

The pituitary is composed of many cells secreting a variety of hormones. We sought to investigate GH promoter methylation in clonally derived cells secreting a single hormone. Therefore, we chose cell types representing GH + (GC cells) and GH − (MMQ cells). Using manual bisulfite sequencing, we determined that the promoter DNA of GH + cells was largely unmethylated (Figure 3A top left panel, open circles) while that from GH − cells was hypermethylated (Figure 3A top right panel, closed circles). Therefore, it is likely that DNA methylation in part restricts GH gene expression in MMQ cells.

In order to assess the role of DNA methylation on transcriptional repression in MMQ cells, we treated cells with a DNA demethylating agent, 5-azacytidine (5-azaC). Examination of mRNA levels revealed that 5-azaC relieved transcriptional silencing of the GH gene (3A, lower panel) with little or no observable change in the transcription of Pit-1 and Prl. Together these results support that 5-azaC can relieve GH repression from a GH – cell line, characterized with a highly methylated DNA promoter.

### The methylated GH DMR recruits a methyl-DNA binding protein

DNA methylation can have profound effects on transcriptional gene regulation by either blocking or recruiting transcription factors [18]. In an attempt to determine how the DMR of GH influences the DNA binding activity of proteins, we used nuclear extracts and a GH DMR probe in electrophoresis mobility shift assays (EMSAs). MMQ cell nuclear extracts were tested for binding to either a methylated or unmethylated radiolabeled probe overlapping the DMR (Figure 3B, left panel). We obtained a strong upper band using nuclear extracts and the methylated probe but not with the unmethylated probe (see arrow, compare lanes 2 and 5). The specificity of binding was confirmed by efficient competition with methylated unlabeled competitor oligonucleotide (lanes 3,6). Next we assessed nuclear protein binding to a methylated radiolabeled probe by using nuclear extracts prepared from different pituitary derived rodent cell lines: MMQ, GC and GHFT. The mobility shift patterns were the same for GC and GHFT nuclear extracts (Figure 3B, right panel, lanes 5–10) while an additional lower band was found using MMQ cells nuclear extracts (lanes 2–4). Furthermore, a methylated unlabeled competitor oligonucleotide competed for binding to the specific upper band (compare lanes 3 and 4, 6 and 7, 9 and 10). Taken together, this suggests that the GH DMR can recruit DNA binding proteins from number of cell lines in a methylation-dependent manner.

The DMR identified in this study contains four CpGs. To identify the methylated CpGs responsible for recruiting a methyl-specific binding protein, either the −8, −7 or −6, −5 were methylated and assessed in an EMSA competition assay (Figure 3C, compare lanes 2–6). Oligonucleotides methylated at positions −8 and −7 efficiently competed for binding while those methylated at positions −6 and −5, did not (compare lane 5 with lane 4). This suggests that the upstream methyl CpGs at positions −8 and −7 are likely sufficient to recruit a methyl-DNA binding protein, in vitro.

Some methyl-binding proteins require methylated cytosines on both DNA strands for binding [19] therefore, we tested if the protein binding was sensitive to upper and lower strand methylation or combinations thereof. Oligonucleotides with each strand individually methylated were not effective competitors (Figure 3C, lanes 8–11). Together, the methyl DNA binding activity described here likely requires symmetrically methylated CpG at positions −8, −7 of the rat GH promoter.

### Characterization of GH DMR binding proteins with competitor oligonucleotides

A number of transcription factors regulating GH expression have been shown to bind within or near the DMR [20], [21]. For example, the thyroid hormone receptor site directly overlaps with the CpG at position −6. A series of competitor oligonucleotides were selected and tested for their ability to compete with the methyl-specific binding activity. Previously these oligonucleotides acted as efficient competitors presumably targeting the following transcription factors: the direct repeat 4, thyroid hormone receptor element (DR4-TRE) for the thyroid hormone receptor, the high affinity Mbox5 for zinc finger E-box binding homeobox 1 (Zeb1) [22], consensus Zeb 1 [23], the GC box for the specificity protein 1 (SP1) (Promega, Part # 9PIE639) family of transcription factors and the high affinity MeCpG site for methyl CpG binding protein 2 (MeCP2) [24]. The result of the competitor EMSA can be found in the lower panel (Figure 3D) while a summary of the results is shown in the Table S1.

The DR4 TRE was an efficient competitor for the lower band (Figure 3D, compare lane 15 with 3). This suggests that the lower band likely represented the binding activity of a protein with similar sequence specificity and DNA affinity as the thyroid hormone receptor. The high affinity Zeb1 binding site, Mbox5, efficiently competed for binding to the band labeled “middle” (compare lane 18 with 3). Interestingly, a consensus Zeb1 binding site was an effective competitor for any of the bands (compare lane 21 with 3). Thus, the DMR oligonucleotide sequence likely contains a high affinity binding site for a protein(s) with similar sequence recognition and affinity as Zeb1. As well, the consensus GC box or SP1 family of protein binding site efficiently competed for binding to the middle band (compare lane 24 with 3). Together these results suggest that the probe used for these experiments contains multiple specific binding sites for proteins independent of DNA methylation.

In an attempt to further characterize the methyl binding activity, we used a high affinity MeCP2 competitor [24]. The results showed that the competitor was not able to displace the upper-methyl-specific binding activity (compare lane 27 with 3), likely ruling out MeCP2 as a candidate methyl DNA binding protein in this context.

### Enrichment of SmcHD1 using methylated GH DMR DNA affinity purification

**Figure 4:**
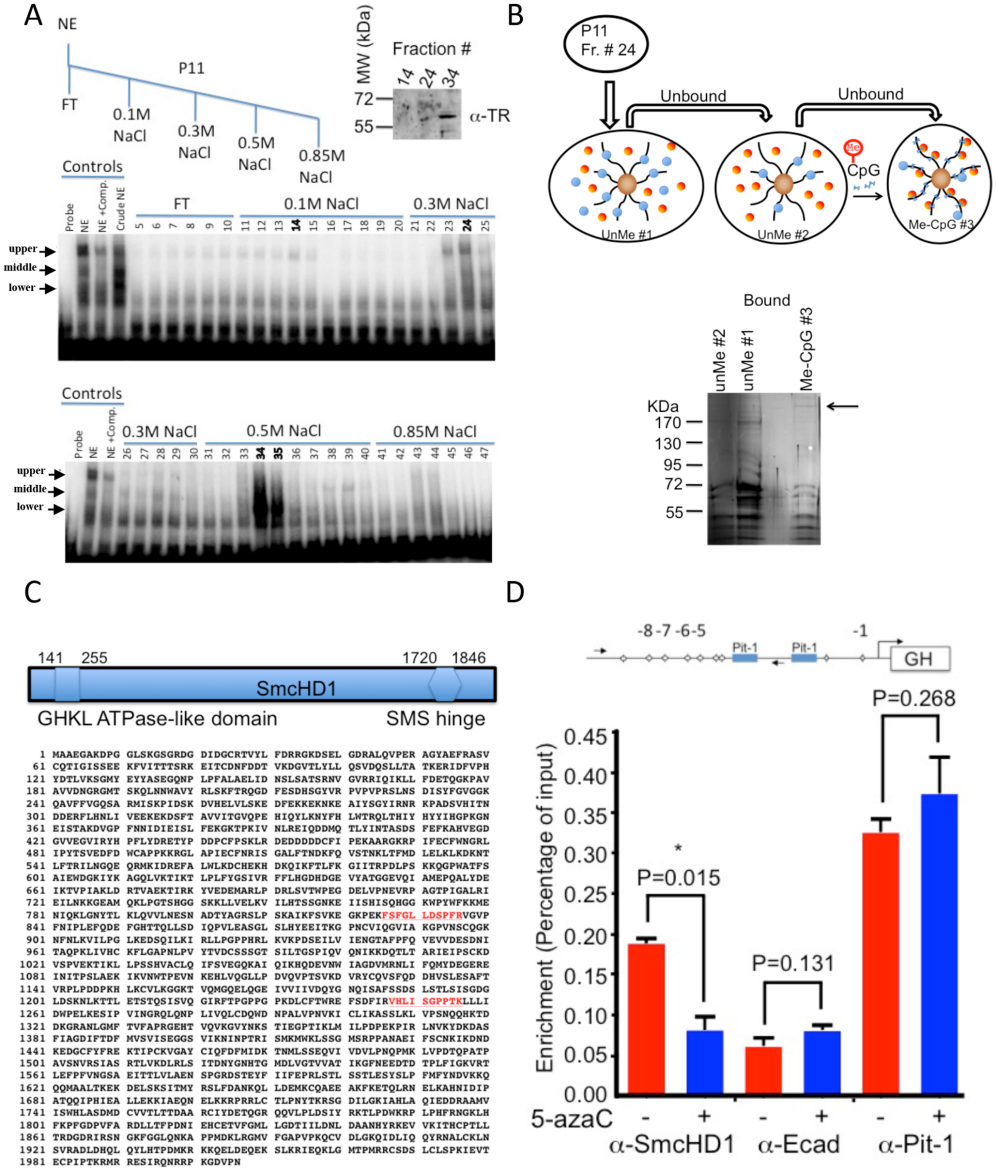
DNA affinity purification of SmcHD1 using a multimerized version of the methylated GH DMR. **A.** Individual DNA binding proteins were separated by fractionation of MMQ nuclear extract. Upper left, schematic description of the step-wise elution of MMQ nuclear extract from a P11 phosphocellulose column. Below, EMSA analysis of individual samples collected from the fractionation. Fractions (#14, #24, and #34) isolated middle, upper and lower DNA binding proteins, respectively. Upper right, immunoblott of selected fractions to detect the presence of thyroid hormone receptor (TR). **B.** Isolation of a protein recruited by the methylated GH DMR. Top, a schematic illustrating the manipulation of the fraction (#24) containing the methyl-DNA specific binding activity. Three columns were generated with the multimerized GH DMR and the third and last one was methylated with SssI methyltransferase. Fraction #24 was applied sequentially to unmethylated columns and then to a third column to capture proteins with affinity to the methylated DNA. Lower, the proteins bound to each of the three columns were resolved on a gradient gel and visualized using a mass-spectroscopy compatible silver stain. A band retained by the DNA methylated affinity column is highlighted by an arrow. The protein was excised from the gel and analyzed by liquid chromatography and mass-spectroscopy. **C.** Identification of SmcHD1 as a molecular component recruited to the methylated DNA. Peptides (in red) matching the molecular mass of amino acid sequences predicted encoding SmcHD1. **D.** Binding of SmcHD1 to the GH promoter is sensitive to cells treated with 5-azaC. Upper, schematic illustration of the GH promoter and relative position of the primers used for the ChIP assays. MMQ cells were treated with 5-azaC or cultured under normal conditions and then processed in a conventional ChIP assays with anti-SmcHD1, anti-Pit1 or anti-E cadherin antibodies. The samples were analyzed by quantitative PCR in triplicate and repeated a minimum of three times. The data is presented as percentage of the input. A Student's t-test was used to assess the statistical differences between untreated and 5-azaC treated cells. P-values are indicated above the sets (* indicates a P-value <0.05).

The EMSAs were unable to provide information on the identity of the DNA binding activity recruited to the methylated version of the DMR. Thus, to identify proteins recruited by the methylated DMR, we devised a protein purification strategy to isolate nuclear proteins using a methyl DNA affinity purification step based on a method described by Yaneva and Tempst [25] (Figure 4A and B upper). Individual fractions of nuclear extracts were collected by salt elution from a P11 phosphocellulose column and tested in an EMSA using a methylated DMR as a probe. The results showed that the middle, upper and lower specific binding activity in that order could be separated using this strategy (Figure 4A). The middle band was eluted using 0.1M NaCl (fraction #14), the upper methyl DNA-binding specific band was enriched by a 0.3M salt elution (fraction #24) and the lower band was highly enriched by the 0.5M salt elution (fraction #34). To provide support for the identity of proteins enriched in those fractions, we used and immunoblot with an anti- thyroid hormone receptor (TR) antibody (Figure 4A, upper right). The results showed that the thyroid hormone receptor was present in the same fraction that was highly enriched with the lower shifted activity (fraction #34). Together these and the competitor EMSA results show strong support that the lower shifted component was likely due to the binding with thyroid hormone receptor.

A single high molecular weight band was the only unique protein band from the DNA affinity column containing the methylated DMR (Figure 4B). Liquid chromatography-mass spectroscopy analysis of the isolated band identified two peptides that matched the mass predicted for amino acids sequences. Both of which were fragmentation products of SmcHD1 polypeptide (Figure 4C and Table S2). Thus, our data suggests that SmcHD1 can be a DMR regulatory protein, in vitro.

### Interaction of SmcHD1 with the GH promoter in cells is sensitive to 5-azaC

To investigate SmcHD1 binding to the GH promoter, we performed a chromatin immunoprecipitation (ChIP) assay with an anti-SmcHD1 antibody in MMQ cells with a preserved DNA methylation state or in cells treated with 5-azaC. The results showed that the anti-SmcHD1 antibody enriched the GH promoter only in untreated cells (Figures 4D and S2). ChIP with anti-Pit-1 antibody was used as a positive control and anti-E-cadherin (E-cad) antibody for estimating background. Together these data support the hypothesis that SmcHD1 can be recruited to the GH promoter in a DNA-methylation dependent manner in cultured cells. We attempted knock-down of SmcHD1 in MMQ cells to demonstrate a direct role for regulation of GH gene expression. However, all attempts to lower the level of SmcHD1 failed in these cells (data not shown). A stable cell line that expressed a Strep-FLAG-tagged SmcHD1 protein in human kidney derived HEK293 cells confirmed that SmcHD1 protein can bind to the GH promoter (Figure S2) and enrichment was lost upon treatment of cells with 5-azaC. These results are consistent with the results of the ChIP experiments in MMQ cells.

### Microarray analysis confirms that loss of SmcHD1 up-regulates X-linked genes

**Figure 5:**
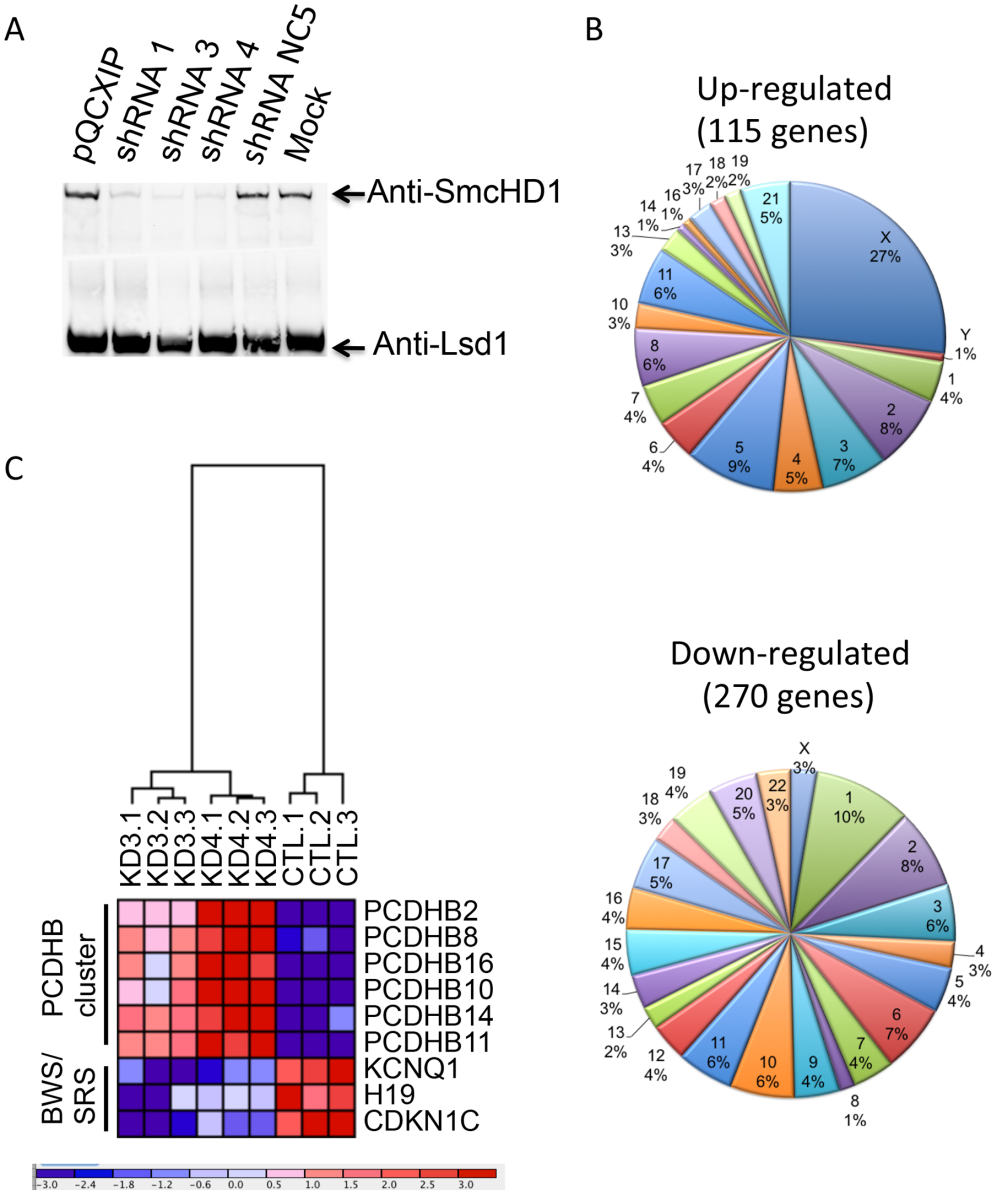
Profiling gene expression from cells with knock-down levels of SmcHD1. **A.** Retroviral shRNA directed towards SmcHD1 efficiently down-regulated SmcHD1 protein levels in HEK293 cells. shRNAs directed towards SmcHD1, a control shRNA or empty plasmid (pQCXIP) was used for retroviral infection HEK293 cells. NEs were prepared from stably infected cells and analyzed by immunoblot with an anti-SmcHD1 antibody. An anti-LSD1 antibody was used as an internal control for loading. **B.** A disproportionate number of genes were up-regulated on the X-chromosome in SmcHD1 knock-down cells. A pie chart was used to illustrate the percentage of genes on each chromosome that were up- or down- regulated in SmcHD1 knock-down cells. **C.** Heat map and hierarchal clustering of selected up- and down- regulated genes in SmcHD1 knock-down cells or cells infected with control non-specific NC5 shRNA. Below, scaling of the fold differences of the genes from cells. Intense red indicates up-regulation and intense blue indicates down-regulation.

In an attempt to further understand the role of SmcHD1 in gene expression, we generated a series of HEK293 cell populations with knock-down levels of SmcHD1 using a short hairpin (sh) retroviral delivery system and performed microarray analysis to determine the influence of reducing SmcHD1 protein level on genome-wide mRNA levels. The knock-down levels of SmcHD1 in these cells was visualized using an immunoblot with whole cell extracts (Figure 5A). shRNA3 and 4 were most effective in knocking-down SmcHD1 levels and thus were used to generate microarray gene expression data comparing the levels of shRNA 3 and 4 infected cells with those infected with a control shRNA, NC5. Triplicates of each data point were generated to produce a P-value and provided a level of confidence in the observed data. Using t-tests between pairs of data sets, those with the P-value <0.05 and an intensity difference greater than 1.8 fold were selected and defined as genes or loci IDs influenced by the loss of SmcHD1. A total of 385 gene “IDs” were identified. Of these, 115 were up-regulated while 270 were down-regulated (Table S3 and S4, respectively). Hierarchical clustering revealed that the expression profile of KD3 (shRNA3) and KD4 (shRNA4) knock-down samples were more similar to each other than to the control samples, CTL (NC5) (Figure S3, tree structure in the top left). Pie charts illustrate the chromosomal distribution of SmcHD1 differentially regulated genes (Figure 5B). As expected the majority of up-regulated genes (27 percent) were localized to the X-chromosome.

### SmcHD1 regulates gene clusters on autosomes

**Figure 6:**
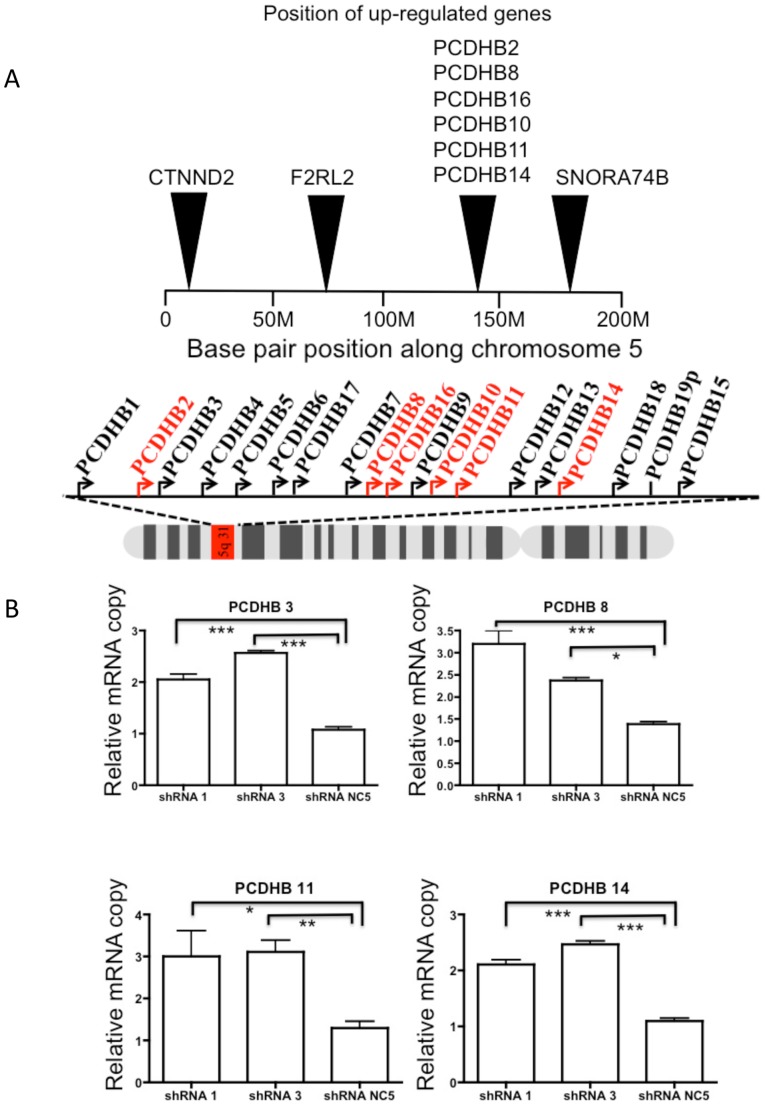
The protocadherin β cluster genes were differentially regulated in SmcHD1 knock-down cells. **A.** A number of genes in the protocadherin β cluster were up-regulated in SmcHD1 knock-down SH-SY5Y cells. A graphical representation showing the position of differentially regulated genes on human chromosome 5. Below in red is the corresponding position of the upregulated genes in the protocadherin β cluster (5q31.3) upon loss of SmcHD1. **B.** mRNA quantitation of selected protocadherin β genes using RT-qPCR in SmcHD1 knock-down SH-SY5Y cells. The copy numbers are relative to and corrected using β-actin cDNA levels. * indicates P-values <0.05, ** P-values <0.01 and *** P-values <0.001 using an unpaired Students t-test.

**Figure 7:**
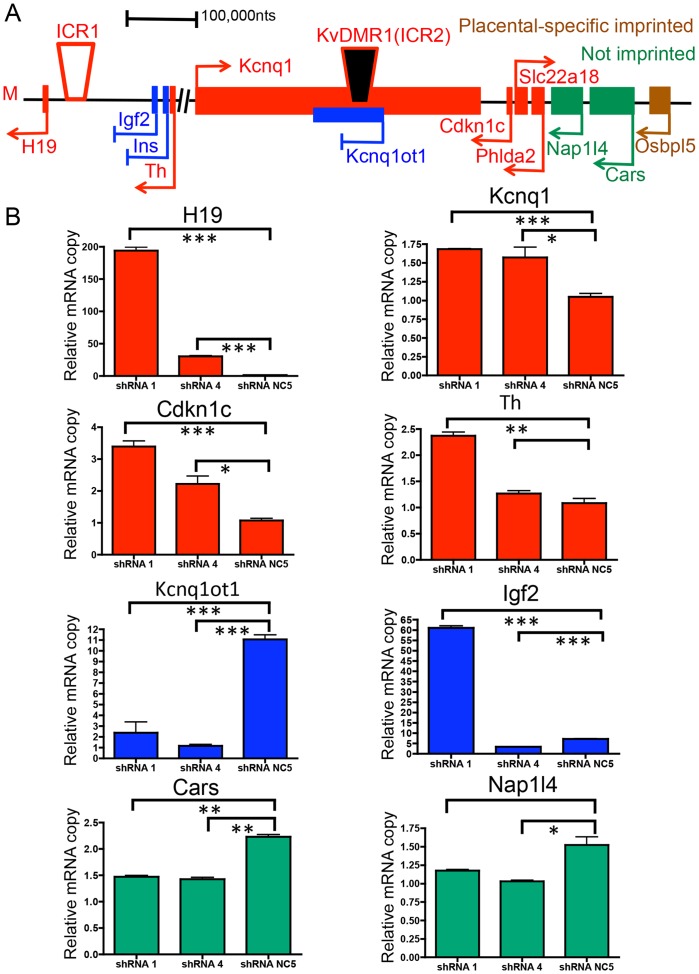
The H19/Igf2 imprinted locus was dis-regulated following SmcHD1 knock-down. **A.** A number of imprinted genes associated with BWS and SRS were dysregulated in SmcHD1 SH-SY5Y knock-down cells. A graphical representation of the H19/Igf2 locus on the human chromosome at position 11p15.5. Maternally imprinted genes are highlighted in blue, maternally expressed genes are in red, one of the placental-specific imprinted genes is colored in brown and not imprinted genes are in green. A non-coding RNA, Kcnq1ot1 is colored in blue and is typically expressed from the paternal chromosome presumably acting to silence genes normally expressed from the maternal chromosome (M) including Kcnq1 and Cdkn1c. Differentially DNA methylated regions (ICR1, KvDMR1 (ICR2)) are indicated by the trapezoids (solid indicates hypermethylation and open hypomethylation). **B.** mRNA quantitation of selected genes in the H19/Igf2 locus using RT-qPCR in SmcHD1 SH-SY5Y knock-down cells. The copy numbers are relative to and corrected using β-actin cDNA levels. * indicates P-values <0.05, ** P-values <0.01 and *** P-values <0.001 using an unpaired Students t-test.

Previous reports suggested that SmcHD1 may regulate gene clusters with monoallelic expression patterns including genes with parent-of-origin monoallelic expression [26], [27]. We identified differentially regulated genes upon loss of SmcHD1 in HEK293 cells that have been previously shown to exhibit monoallelic expression (Table S5). We found two clusters of genes, including members of the protocadherin β gene cluster and imprinted genes associated with BWS/SRS including: potassium voltage-gated channel KQT-like subfamily member1 (Kcnq1); H19 and cyclin-dependent kinase inhibitor 1C (Cdkn1C). The expression of those gene sets are illustrated in a heat map using hierarchical clustering (Figure 5C). Upon knock-down of SmcHD1, all differentially regulated genes of the protocadherin β cluster were up-regulated (Figure 6A, red). We tested knock-down of SmcHD1 in a more relevant cell line SH-SY5Y cells, a neuroblastoma derived cell line. RT-qPCR confirmed that PCDHB 3, 8, 11 and 14 were significantly up-regulated following SmcHD1 knock-down (Figure 6B). To determine if DNA methylation was altered, we performed bisulfite pyrosequencing on the PCDHB 10 promoter but DNA methylation was not significantly changed (Figure S4). Overall, these results suggest that through an unknown mechanism, SmcHD1 may repress gene expression of some members of the protocadherin β cluster. Conversely, three genes associated with BWS/SRS (Kcnq1, H19 and Cdkn1c) are positioned in a cluster at chromosome 11p15.5 and are down-regulated in SmcHD1 knock-down HEK293 cells (Figure 5C and 7A). Typically these genes are expected to be expressed from the maternal allele and are silenced on the paternal chromosome [28]. Genes in this locus are regulated through two differentially methylated regions, KvDMR1 (imprinting control region 2 (ICR2)) and ICR1. The results showed that in SH-SY5Y cells genes typically expressed only on the maternal chromosome were up-regulated in knock-down cells (H19, Kcnq1, Cdkn1C) while those typically imprinted (silenced) on the same chromosome were further repressed including the non-coding Kcnq1ot1 (Figure 7B). The effects on tyrosine hydroxylase (Th) and insulin-like growth factor 2 (Igf2) expression are unclear. In addition, two non-imprinted genes, nucleosome assembly protein 1-like 4 (Nap1l4) and cysteinyl-tRNA synthetase (Cars) were down-regulated upon knock-down of SmcHD1. In summary, the imprinted cluster associated with BWS and SRS was dysregulated in SmcHD1 knock-down HEK293 and SH-SY5Y cells.

## Discussion

In this study, we demonstrated that SmcHD1 binds to the GH promoter and is likely a new regulatory protein for GH gene expression. Further, we identified a GH promoter DMR and characterized the binding of a methyl DNA binding protein to the methylated DMR. In addition, we show that SmcHD1 can repress the protocadherin β gene cluster and extends current findings to regulation of a second imprinted gene cluster associated with BWS/SRS.

### SmcHD1 is involved in regulation of the GH gene

GH gene regulation in the pituitary provides an excellent model to study the effects of promoter DNA methylation in a non-CpG island context. GH plays a central role in modulating growth of some organisms from birth until puberty. In humans, GH secretion declines by 14% per decade in adulthood, with GH deficiency frequently occurring beyond 60 years of life [29]–[31]. GH hormone replacement therapy used in GH deficient individuals can increase muscle mass and strength, bone mass and quality of life while decreasing fat mass [2]. Somatotrope cells secrete GH, the predominant cell-type in the pituitary. The GH secreting lineages as well as two others, secreting Prl and TSHβ, lactotropes and thyrotropes, respectively, emerge from anterior portion of the pituitary. The identity of these cells is established by expression of a functional Pit-1 protein. Understanding mechanisms that govern GH regulation is useful to provide insight into growth and age related changes in GH production.

The GH gene is regulated by distal elements that are positioned over 13,000 bases upstream of the GH transcriptional start site. These include an evolutionarily conserved putative LCR that was originally characterized using human chromosome fragments and transgenic reporter genes in mice [15]. The distal LCR was defined as pituitary-specific DNaseI hypersensitive sites concomitant with gene expression. Interestingly, Lunyak et al. characterized a chromatin boundary element near the LCR using recombined human BAC transgenes in mice [32]. The proximal promoter has cis-regulatory elements located in the first 320 bp upstream of the transcriptional start site [33]. This minimal cassette, in the absence of the surrounding DNA sequences and chromatin is sufficient to drive expression of GH in somatotropes and repression or silencing in lactotropes using randomly inserted mouse transgenes [5]. However, in the presence of surrounding DNA and chromatin, the LCR is absolutely required for the expression of the GH gene.

Analysis of promoter CpGs using wild-type mice was not informative for identification of CpGs that were unmethylated and crucial for gene expression because most promoter CpGs were methylated to the same degree (Fig. 2A and B). Here, we show that BAC recombined transgenes are useful for identification of CpG sites that can bind to regulatory proteins. Using this model, we identified three promoter CpGs that were hypomethylated (−8, −7, −6) and was defined as the GH DMR (Figure 1).

The methylated version of the GH-DMR recruits a methyl-DNA binding protein (Figure 3B). We identified SmcHD1 as a DMR DNA binding protein in vitro (Figure 4C). In cells, we determined that SmcHD1 was bound to the methylated DNA preserved GH promoter and was dismissed upon pretreatment of cells with 5-azaC (Figure 4B). This is the first example demonstrating that SmcHD1 is a component of the machinery that could regulate GH gene expression.

SmcHD1 plays a role in the maintenance of DNA methylation on the inactive X-chromosome (Xi) and is essential for female viability [34]. During development, the Xi acquires CpG island methylation in two phases: a rapid and late phase. The late phase is SmcHD1 dependent [35]. In cells, the Xi forms a compact structure referred to as barr body. SmcHD1 and HBiX1, a heterochromatic protein 1 (HP1) binding protein, together act to link the trimethylated histone H3 lys9 (Me3H3K9) and XIST- trimethylated histone H3 Lys27 (Me3H3K27) chromatin domains to organize the compact Xi structure [36], suggesting a role for SmcHD1 in organizing chromatin domains.

In humans, a deletion mutation (K274del) in SmcHD1 located at a conserved residue in the GHKL ATPase domain combined with a permissive 4q35 allele was suggested as a cause for Facioscapulohumeral muscular dystrophy type 2 (FSHD2) [37]. An autosomal dominant version of the disease mapped to 4q35 or more specifically a D4Z4 repeat. Whole exome sequencing of individuals from seven unrelated family members with FSHD2 identified heterozygous out-of-frame and missense mutations as well as splice variants of SmcHD1 (79% of those tested) [38]. In addition, ChIP analysis revealed that SmcHD1 bound directly to the D4Z4 metastable repeats with each repeat containing an open reading frame encoding double homeobox 4 (DUX4) [38]. Moreover, individuals with the autosomal-dominant FSHD type I form of the disease have at least one allele with 1–10 copies of a D4Z4 repeat. Typically, the general population has between 11–150 repeats further demonstrating the repeat is highly polymorphic. Families with 8 to 10 copies of the repeat were affected by SmcHD1 mutation, suggesting that it is a modifier of the type I form of disease [39].

### SmcHD1 is involved in regulation of genes on autosomes

Homozygous SmcHD1 mutation was lethal in male mice with mixed backgrounds suggesting SmcHD1 is essential for regulation of genes on autosomes [27]. Our results support a major biological role for SmcHD1 in mediating silencing on the inactive X chromosome (Figure 5B). By mapping the position of differentially regulated genes to autosomes, we also found an unusual number of genes that have been characterized as imprinted or having monoallelic expression profiles (Figure 5C and Table S5).

### The role of SmcHD1 in regulation of genes associated with BWS and SRS

BWS/SRS are reciprocal diseases, characterized by dysregulation of a cluster of genes with parent-of-origin imprinting. We identified imprinted genes belonging to the H19/Igf2 and Kcnq1 locus located on chromosome 11p15 (Fig. 5C and Table S5). This region has been shown to be associated with forms of SRS and BWS syndromes. BWS cases are sporadic and are characterized by different imprinting defects including defects in the ICR1 region that results in loss of imprinting of Igf2 and hypermethylation of H19 and in most cases loss of methylation of the KvDMR1 that leads to gain of the non-coding Kcnq1ot1 transcript and loss of Kcnq1 and Cdkn1c expression [40]. Similarly, most SRS cases are also sporadic and are characterized by loss of methylation at the ICR1 locus and gain of expression of H19 at the expense of Igf2 expression [41]. In a more relevant cellular model, SH-SY5Y neuroblastoma cells, knock-down of SmcHD1 displayed dysregulation of BWS and SRS associated genes. All three genes (Kcnq1, H19 and Cdkn1c) found down-regulated in HEK293 cells were up-regulated in SH-SY5Y cells (Figure 7B). Differences in chromatin organization may account for distinct gene expression outcomes observed between the two cell lines. Th and Igf2 genes have mixed results, while non-imprinted genes in the locus, Nap1l4 and Cars, were down-regulated upon SmcHD1 knock-down. Interestingly, the non-coding transcript Kcnq1ot1 was down-regulated. This data supports a model in which the locus adopts imprinting patterns typical of the maternal chromosome as observed in patients with SRS. Determining gain or loss of nucleotide diversity in the mRNAs would conclusively demonstrate if biallelic expression of genes were encoded by the H19/Igf2 locus following knock-down of SmcHD1.

In this study, we present evidence that SmcHD1 is important for regulation of genes on the inactive X-chromosome and non-sex chromosomes in human cell lines. Identification of genes associated with BWS and SRS extends our understanding of the role of SmcHD1 in regulating imprinted clusters beyond that associated with Prader-Willi syndrome (PWS) and Angelman syndrome (AS) [26], [27]. BWS and SRS are congenital disorders with opposite outcomes on prenatal and postnatal growth: gigantism and dwarfism, respectively [42]. BWS is characterized by three major features: overgrowth, macroglossia and anterior abdominal wall defects from diastasis recti to exomphalos [43]. The incidence of BWS is 1/13,000 [44] representing 300 births per year in the United States. SRS is characterized by intrauterine growth restriction and characteristic facial features [45]. Meta-analysis for tumor risk determined that 13.7% of individuals afflicted with BWS developed tumors [46]. The majority were Wilms tumors, hepatoblastomas, rhabdomyosarcomas and neuroblastoma. Thus, SmcHD1 is a key regulatory protein for correct expression of imprinted genes.

### SmcHD1 represses the expression of genes from the protocadherin β cluster

Protocadherins are involved in mediating cell-to-cell contact/signaling, especially in human neuroblastoma cells [47]. An eleven-zinc finger protein (CTCF) in cooperation with the cohesion complex, which contains structural maintenance of chromosomes 3 (Smc3) protein play significant roles in monoallelic and combinatorial expression of protocadherin genes required for proper neuronal differentiation [48]. Previous studies showed that SmcHD1 regulates clustered protocadherin genes (α, β and γ) in mouse embryos [26], [27]. For the first time we showed in human cells that SmcHD1 also regulates expression of protocadherins β genes but this may be limited to the β cluster (Figure 5C and 6). Some studies demonstrated genes of the α and γ cluster were expressed from only one allele [49]. Moreover, the protocadherin β cluster has been shown to adopt monoallelic and combinatorial expression in Purkinje cells in mouse brain [50]. Only a subset of protocadherins are expressed in each neuron and only from one allele selected randomly. However a number of protocadherin β genes are activated in SmcHD1 knock-down cells, therefore SmcHD1 may play important roles in defining molecular events governing neuronal networks.

### Future Directions

In the future, identification of proteins associated with SmcHD1 will provide additional information on the role of SmcHD1 in chromatin-mediated events. In addition, it will be interesting to explore whether SmcHD1 is directly required for the establishment or maintenance of monoallelic gene expression and whether the up-regulation of genes or non-coding RNA (i.e. H19) was due to a switch from mono- to bi-allelic expression.

## Materials and Methods

### Ethics Statement

This study was carried out in strict accordance with the recommendations in the Guide for Care and Use of Laboratory Animals of the National Institutes of Health. The protocol was approved by the Institutional Animal Care and Utilization Committee of the University of California, San Diego.

### Cells, Antibodies and Reagents

Cell lines used in this study were: MMQ (ATCC, CRL-10609), GC [51], GHFT [52], HEK293T (ATCC, CRL-11268) and SH-SY5Y (ATCC, CRL-2266). Antibodies used in this work included: anti-GH, anti-Prl (prolactin), anti-TSHβ, anti-Pit-1 (1769), anti-TRα/β (Santa Cruz, sc-772, fl-408), anti-FLAG (Sigma, Cat. # F3165), anti-E-cadherin (Santa Cruz, sc-7870, H-108) and anti-SmcHD1 antibody (Abcam, Cat. # ab31865). 5-Azacytidine was purchased from Invitrogen.

MMQ cells from rat pituitary were cultured in Ultraculture media (Biowhittaker, Cat. #12-725F) without L-glutamine and supplemented with 5% fetal bovine serum (FBS) and were maintained in a humidified atmosphere containing 5% CO_2_ at 37°C. HEK293T, SH-SY5Y and GHFT cells were cultured in Dulbecco's Modified Eagle's Medium (DMEM; Gibco) containing 4.5 g/L Glucose and L-Glutamine (Biowhittaker, Cat. # 12-604F) and supplemented with 10% fetal bovine serum (FBS). GC cells were cultured in Dulbecco's Modified Eagle's Medium (DMEM; Gibco) containing 4.5 g/L Glucose and Glutamax (Invitrogen, Cat. # 10566-016) supplemented with 12.5% horse serum and 2.5% FBS. Where indicated, cells were treated with 0.25% 5-azaC every 24 hrs for a minimum of 72 hrs.

### BAC recombination

WT GH:RFP BAC plasmids have been described elsewhere [32]. The mouse BAC 418O11 (Genbank accession number: AL604045.7) from the RPCI-23 library (http://bacpac.chori.org/) was used for recombination and contains 221.4 kb from chromosome 11 (nts:106,217,952–106,439,350, build Mouse Dec. 2011 (GRCm38/mm10) Assembly). This region includes 137.5 kb upstream of the major GH transcriptional start site to 82.3 kb downstream of the 3′UTR. The region deleted from the LCR BAC removed sequences −14,891 to −14,723 upstream of the GH transcriptional start site. The mouse GH promoter (starting at −316) and gene ending at nt +1,715 (downstream of the GH transcriptional start site) in the GH 3′ UTR were replaced by the homologous rat GH promoter and the coding sequence for DsRed derived from (Genbank accession number: AF506026). The sequence replaced included the rat promoter sequence −308 to +6 nts (chromosome 10: 95,694,111 −95,694,425, build Rat Nov. 2004 [Baylor 3.4/rn4]) fused to the DsRed through a short linker (127 bp) containing a sequence derived from the human GH-5′ UTR, 1st exon and the first intron (chromosome 17: 61,996,064–61,996,190, build Human Feb. 2009 (GRCh37/hg19) Assembly) beginning at position +8 of the human GH1 transcript. The splice between the human sequence with the DsRed encoding sequence was designed in a way that the initiating codon would begin with the DsRed coding sequence.

The LCR deletion was created in a similar way as described for the SineB2 deletion [32]. Briefly, 50 bp arms flanking the deleted region was used to amplify a galactose kinase (GalK) gene by PCR and the product electroporated into the rGH:RFP BAC containing SW102 E.coli strain. A recombination event was selected using minimal media plates containing galactose as the sole sugar source. Colonies positive for a recombination event (GalK ΔLCR rGH:RFP) into the desired locus were verified by PCR and Southern analysis and then induced for a second recombination event. Synthetic oligonucleotides with the same arms but lacking the intervening GalK gene were electroporated into the GalK rGH:RFP containing BAC SW102 strain. The recombination event was negatively selected on 2-deoxy-galactose (DOG) containing minimal plates for elimination of the selectable GalK gene. The recombination event was verified using PCR and the integrity of the BAC confirmed using DNA fingerprinting. Finally, the LCR deletion was confirmed by sequencing the PCR product using oligonucleotides flanking the area targeted for deletion.

The BAC transgenes were amplified, purified and microinjected into 2–4 cell stage embryos in the University of California, San Diego transgenic core facility. Pup tail ends were genotyped with oligonucleotides recognizing the rGH:RFP component of the BAC. Those positive for the recombined BAC were bred and confirmed for germ line transmission and further propagated as individual mouse lineages.

### Pituitary imaging and Tissue immunofluorescence

Fresh mouse pituitary and hindbrain tissue were carefully dissected and directly imaged using a rhodamine filter and then bright field microscopy. Fixed tissue sections were labeled with antibodies to GH, Prl or TSHβ and FITC and visualized with Cy2 conjugated secondary antibodies (Jackson Labs). Images were captured using a rhodamine and FITC filters.

### Manual bisulfite sequencing and CoBRA

Following dissection of pituitaries or harvesting of cells, genomic DNA was prepared using the Qiagen Blood & Cell culture kit (Qiagen, Cat. # 13323) and stored at −20C. Genomic DNA was treated with bisulfite as previously described [53] or in other instances using the Imprint DNA Modification Kit (Sigma, Cat. # MOD50-1KT). The DNA was then amplified by PCR and products were cloned using the Stratagene pGEM T-easy kit (Promega, Cat. # A1360). A number of individual colonies were selected and prepared for sequencing (Eurofins MWG Operon). The sequencing results were analyzed using the quantification tool for methylation analysis (QUMA, http://quma.cdb.riken.jp/) for statistical analysis.

For combined bisulfite restriction analysis (CoBRA), the PCR samples were digested with FokI to reveal the ratio of unmethylated vs methylated Cs. FokI cuts bisulfite modified DNA (C→T), unmethylated. The oligonucleotides used for amplification are listed in (Table S6).

### Reverse transcription quantitative PCR

RNA was prepared using Trizol (Life Technologies, Cat. # 15596018) according to the manufacturer's protocol. Approximately, 100ng of RNA was reverse-transcribed using Superscript II (Life Technologies, Cat. # 18064-014) according to the manufacturer's protocol. The cDNA was quantified using SYBR Advantage qPCR Premix (Clontech, Cat. # 638320), Rox as an internal standard and a StepOne Real Time PCR System (Life Technologies). For quality control, the melting curve for each primer set was verified and the PCR product ran on a agarose gel for detection of a single band of the expected length. The oligonucleotides are listed in Table S7. Following qPCR, the threshold levels were adjusted manually to logarithmic part of the curve to obtain a Ct value. The Ct values were normalized with those obtained by quantitation of the β-actin message and then the relative mRNA levels were determined. Where appropriate the data was analyzed using a Student's t-test and the level of confidence displayed as P-values.

### SDS-PAGE and immunoblot

Denatured proteins samples were separated on sodium dodecyl sulfate-polyacrylamide (SDS-PAGE) gels. DNA affinity purified proteins were run on a gradient gel (Invitrogen tris-tricine based buffer system) and were visualized using a mass-spectroscopy compatible silver stain kit (SilverQuest, Invitrogen, Cat. # LC6070) according to the manufacturer's protocol.

For preparation of whole cell extracts (WCE), cells were pelleted and washed one time with PBS then lysed in one pellet volume of RIPA buffer (50 mM Tris-HCl pH 7.4, 150 mM NaCl, 1 mM EDTA, 1% Sodium deoxycholate, 0.1% SDS) supplemented with a protease inhibitor cocktail (Bioshop, Cat. # PIC003). The re-suspended samples were placed on ice for 20 minutes, vortexed and then centrifuged for 5 min at maximum speed (14,000 xg). The protein supernatant was quantified and equal quantities diluted in sample buffer and boiled for 5 minutes. Proteins were separated on 6% SDS-PAGE acrylamide gels using a Tris-acetate buffering system [54] then transferred to nitrocellulose membranes (Millipore, Protran BA85). After transfer, membranes were blocked in 0.05% milk powder in PBS containing 0.01% Tween-20 then incubated overnight in 1∶1000 primary antibody. After washing in PBS+0.01% Tween-20, the membranes were incubated with secondary HRP antibody (Jackson Labs, 1∶50,000 dilution), developed using SuperSignal West Dura Extended Duration Substrate (Thermo Scientific, Cat. # 37071) and visualized using a cooled CCD instrument (Dyversity, Syngene).

### Chromatin Immunoprecipitation Assay

Chromatin immunoprecipitation (ChIP) was performed as previously described [55]. The cells or pituitary tissue were fixed using 1% formaldehyde in HEPES (pH 7.8) for 10 min at room temperature. Cells were collected, washed with PBS, re-suspended in lysis buffer (50 mM Tris-HCl, pH 8.1, 1% SDS and 10 mM EDTA) and sonicated using a Branson Sonifier 450 with an output of 3.5 and constant duty cycle in pulses until the cross-linked DNA fragments were 300–500 bp in size. Five percent of the cross-linked chromatin was used as input and the rest was incubated with 5 µg of poly deoxyinosinic-deoxycytidylic (poly dI-dC) and either 40 µL of 50% slurry of anti FLAG affinity gel (Sigma) or a primary antibody as indicated overnight, at 4°C. For the primary antibody samples, Protein A agarose beads were added for an additional 20 minutes prior to washing. The beads were successively washed with RIPA (10 mM Tris-HCl pH 8.0, 1 mM EDTA, 0.5 mM EGTA, 140 mM NaCl, 1% Triton-X 100, 0.1% Na-deoxycholate, 0.1% SDS and 1X protease inhibitor cocktail (Bioshop, Cat. # PIC003)), TSEII (20 mM Tris-HCl pH 8.1, 500 mM NaCl, 2 mM EDTA, 0.1% SDS, 1% Triton X-100), TSE III (10 mM Tris-HCl pH 8.1, 0.25 M LiCl, 1 mM EDTA, 1%NP-40, 1% sodium deoxycholate) buffer and then 3 washes with 0.1X TE. The DNA crosslinks were reversed overnight at 65°C in 0.1 M NaHCO_3_. The DNA was then precipitated with 2 µL of pellet paint (Novagen), 1/10 volume 3M Na-acetate and 2 volumes of 100% EtOH followed by centrifugation for 10 min at 14,000 rpm. Pellets were washed with 70% EtOH, dried and re-suspended in 50 µL ddH2O. For each PCR reaction 5 µL of the immunoprecipitated sample or five percent input samples were used to quantify enrichment.

### Preparation of nuclear extract and electrophoretic mobility shift assay (EMSA)

Nuclear proteins from MMQ, GHFT and GC cells were prepared as previously described [56]. Briefly, the cells were washed twice with ice cold PBS followed by incubation on ice for 10 min in excess of buffer A (10 mM HEPES-KOH pH 7.9, 1.5 mM MgCl2, 10 mM KCl, 0.5 mM DTT, and protease inhibitor cocktail). Cells were lysed by vortexing and the nuclei were collected by centrifugation at 4400 rpm for 15 min. The pellets were re-suspended with 1 pellet volume of Buffer C (20 mM HEPES-KOH pH 7.9, 1.5 mM MgCl2, 420 mM NaCl, 0.2 mM EDTA, 0.5 mM DTT, 25% glycerol, and 1X protease inhibitor cocktail) and incubated on ice for 20 min and then centrifuged at >15000 rpm. The supernatants containing the nuclear proteins were collected and stored at −80°C.

For radiolabelling oligonucleotides with α^32^P-dCTP, 7 pmol of double stranded oligonucleotides were filled-in with T4 DNA polymerase (Klenow). The labeled probes were separated from the free nucleotides using Illustra Microspin G-50 columns (GE healthcare). The oligonucleotide sequences used in the EMSAs and competition assays are provided in the Table S1.

For the electrophoretic mobility shift assay (EMSA), the binding reactions were prepared using 12 µg of nuclear extracts in binding buffer (10 mM TRIS–HCl pH 7.0, 1 mM DTT, 5 mM MgCl2, 50 ng/mL poly-dIdC, 2.5% glycerol, 0.05% Igepal, 0.05 M KCl and 50,000 cpm of labeled oligonucleotide). The reactions were incubated at room temperature for 20 min followed by separation using 4% non-denaturing polyacrylamide gels. For competition assays, cold/unlabeled competitors were added at 100 fold molar excess. Gels were dried and exposed to a phosphor storage cassette in the dark for 12 hrs followed by scanning using Molecular Dynamics Storm 860 Phosphor imager.

### Large-scale protein purification

Nuclear extracts were prepared from 4×10^10^ MMQ cells and applied to a P11 column, pre-washed with PBS with no salt and then proteins were eluted in a step-wise fashion with PBS containing (0.1 M NaCl, 0.3 M NaCl, 0.5 M NaCl and finally 0.85 M NaCl) and collected in 1 mL fractions. Methyl-DNA binding proteins were monitored using a standard EMSA with a methyled DMR oligonucleotide probe. Next, the methyl DNA binding activity concentrated in fraction #24 was applied to a DNA affinity column containing a mulitmerized unmethylated version of the DMR. The flow-through was added sequentially to a second unmethylated column and then finally to a third version that was DNA methylated. The bound proteins were washed with PBS, 0.01% NP-40 and eluted in SDS-sample buffer prior to gel electrophoresis. To generate the affinity matrix, an oligonucleotide with 2 copies of the DMR (RH75) was amplified in a PCR reaction with a second oligonucleotide containing a biotin group at the 5′ end (GP816) (Table S8). The PCR product was qualified on an acrylamide gel and the DNA methylated oligonucleotides prepared by treatment with SssI methyltranferase (NEB). The methylated version of the column was confirmed by digestion with the methylation sensitive HgaI restriction enzyme (Figure S5).

### Short hairpin knock-down of SmcHD1 in cells

Double-stranded nucleotide sequences encoding shRNAs targeting SmcHD1 and a control NC5 sequence were cloned into the pQCXIP-GFP vector (Table S9) [57]. For production of shRNAs expressing virus particles, HEK293T cells were co-transfected with the individual shRNA expression vectors and the packaging plasmid (pCL10A1) using Polyfect (Qiagen) according to the manufacturer's protocol. The media from transfected cells were collected after 48 and 72 hours and combined with 10 µL/mL Polybrene (2 mg/mL in 1 M HEPES pH 7.2). Then the virus stock was passed through a 0.2 µm syringe filter and stored at 4°C.

HEK293 or SH-SY5Y cells were infected two times in a sequential fashion. Briefly, 2 mL of virus stock was added to cells seeded on 6 well plates and the plates were centrifuged for 1.5 hrs at 2500 rpm and 30°C and then the media was replaced with fresh DMEM complete media. The procedure was repeated the following day. Two days after the start of the infection process, cells were selected with 3 µg/mL of puromycin for 7 days. For the SH-SY5Y cells, the selection was initiated 1 day post-infection and harvested after 11 days post-infection. The infection was monitored using GFP expression.

### mRNA Microarray analysis

The mRNA from stable cell lines was isolated using TRI reagent (Sigma, Cat. # T9424) according to the manufacturer's protocol. The mRNA microarray was performed by the laboratory for Advanced Genome Analysis at the Vancouver Prostate Centre, Vancouver, Canada. Total RNA was qualified with the Agilent 2100 Bioanalyzer (RNA) and quantified with the NanoDrop ND-1000 UV-VIS spectrophotometer to measure A260/280 and A260/230 ratios. The RNA was converted to cDNA using T7 RNA polymerase in the presence of cyanine 3 (Cy3)-labeled CTP. Samples were prepared in biological triplicates following Agilent's One-Color Microarray-Based Gene Expression Analysis Low Input Quick Amp Labeling v6.0. An input of 100 ng of total RNA was used to generate Cyanine-3 labeled cRNA. Samples were hybridized on Agilent SurePrint G3 Human GE 8×60K Microarray (Design ID 028004).

Arrays were scanned with the Agilent DNA Microarray Scanner at a 3 µm scan resolution and data was processed with Agilent Feature Extraction 10.10. Green processed signal was quintile normalized with Agilent GeneSpring 11.5.1. To find significantly regulated genes, fold changes between the SmcHD1 shRNAs and the NC5 shRNA control groups and P-values gained from t-test between the same groups were calculated with a Benjamini-Hochberg multiple testing correction. The t-tests were performed on log transformed normalized data and the variances were not assumed to be equal between sample groups. Up and down-regulated genes were selected if the P-value was <0.05 and fold difference greater or equal to 1.8 compared to the control. The raw data was submitted to the GEO repository, GSE52065.

Heat maps were created using the Hierarchical clustering program from the GenePattern website (http://genepattern.broadinstitute.org). To map the genes to chromosomal locations, we used the biomart program located at http://uswest.ensembl.org. The Ensemble Genes 70 and *Homo sapiens* genes (GRCh37.p10) were chosen as databases for analysis. Selected genes from the microarray analysis were mapped on chromosomes by filtering using the Agilent Sureprint G3 GE 8x60k probe's IDs.

## Supporting Information

Figure S1 (TIF)

Figure S2 (TIF)

Figure S3 (TIF)

Figure S4 (TIF)

Figure S5 (TIF)

Table S1 (TIF)

Table S2 (TIF)

Table S3 (XLS)

Table S4 (XLS)

Table S5 (TIF)

Table S6 (TIF)

Table S7 (TIF)

Table S8 (TIF)

Table S9 (TIF)
