# Supplementary figures and images for: Epigenetic Characterization of the Growth Hormone Gene Identifies SmcHD1 as a Regulator of Autosomal Gene Clusters
Source: PLoS One. 2014 May 12;9(5):e97535. doi: 10.1371/journal.pone.0097535 (PMC4018343; doi:10.1371/journal.pone.0097535)

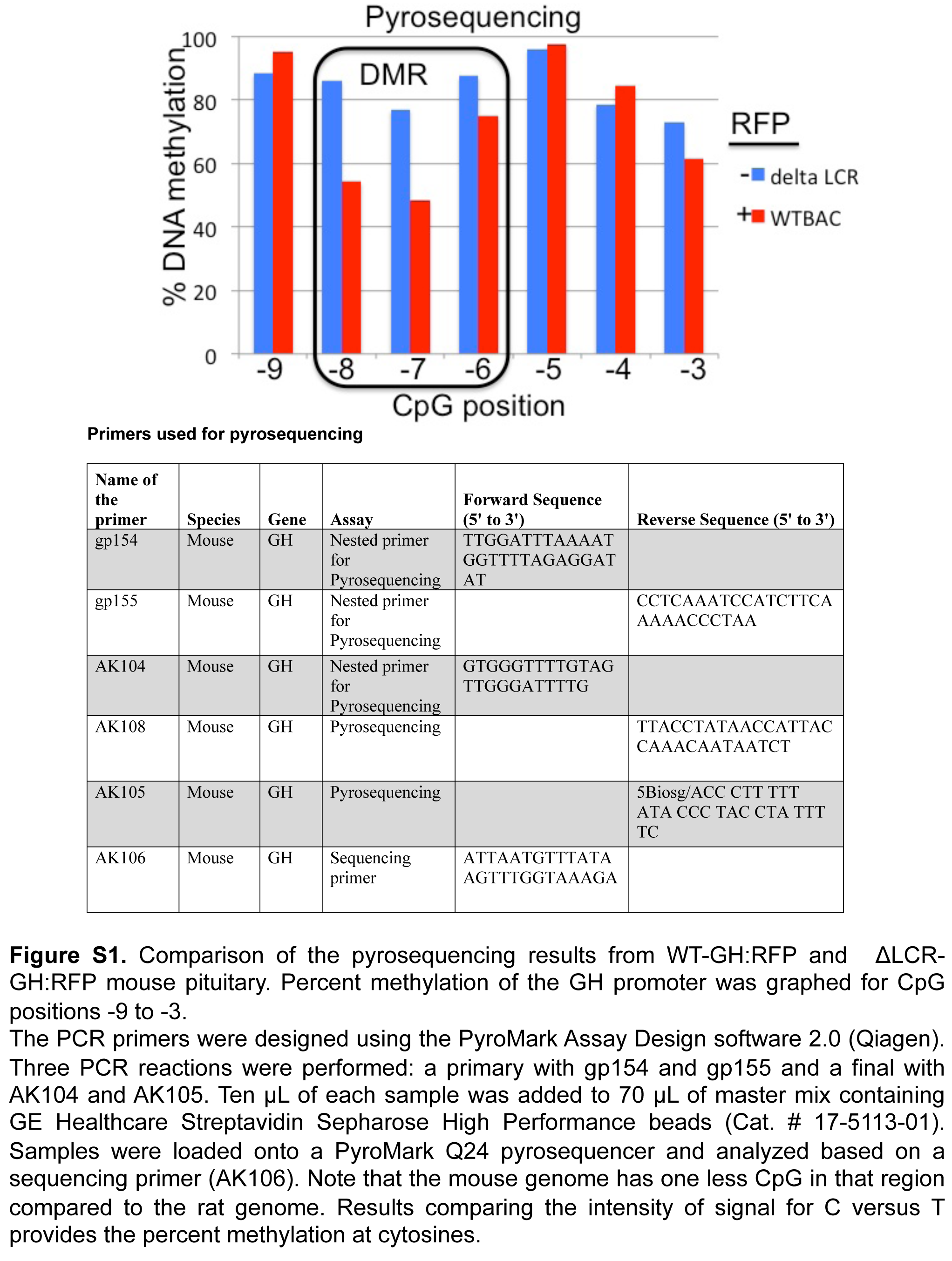

Supplement: Figure S1 — (TIF) [file pone.0097535.s001.tif]

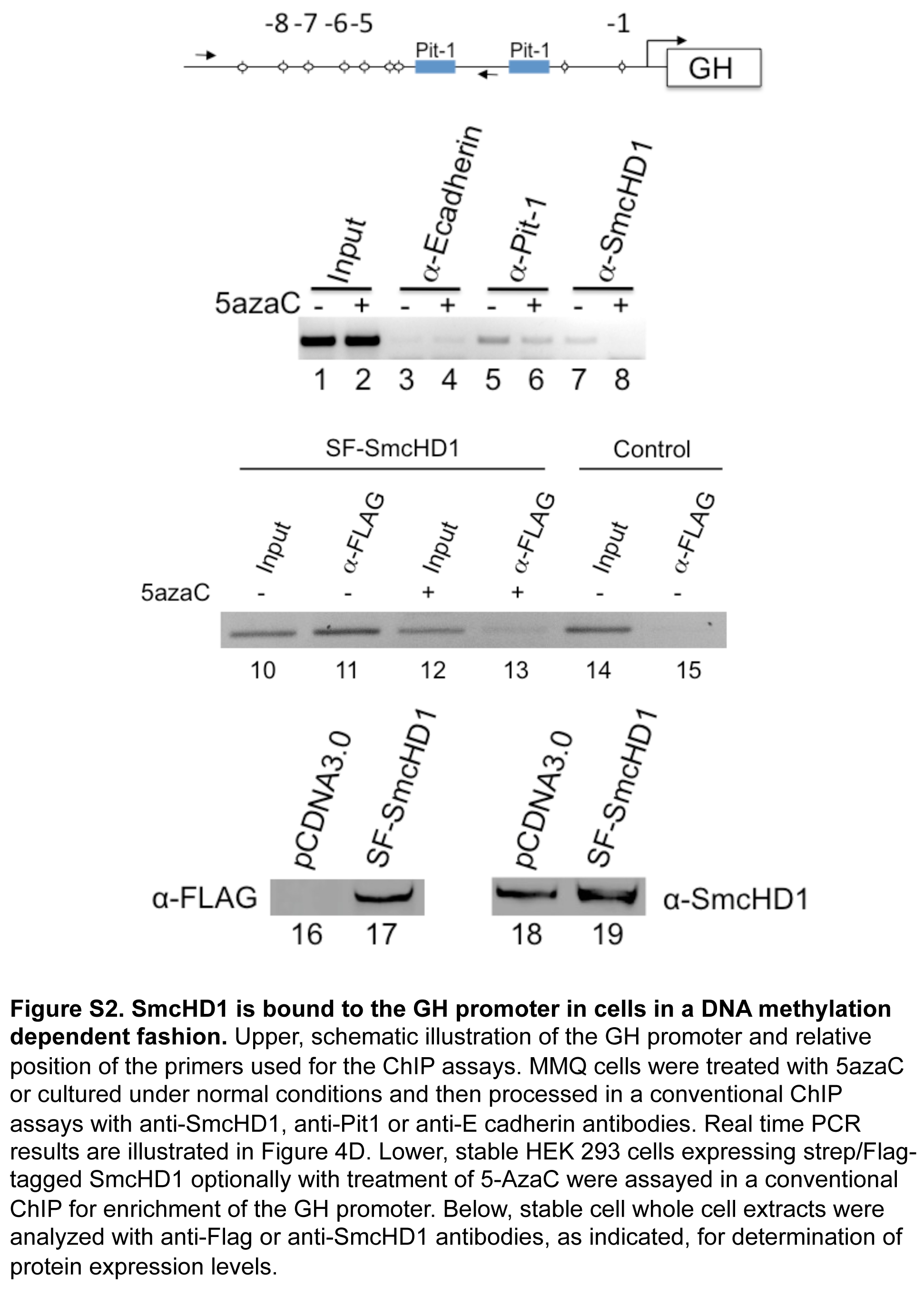

Supplement: Figure S2 — (TIF) [file pone.0097535.s002.tif]

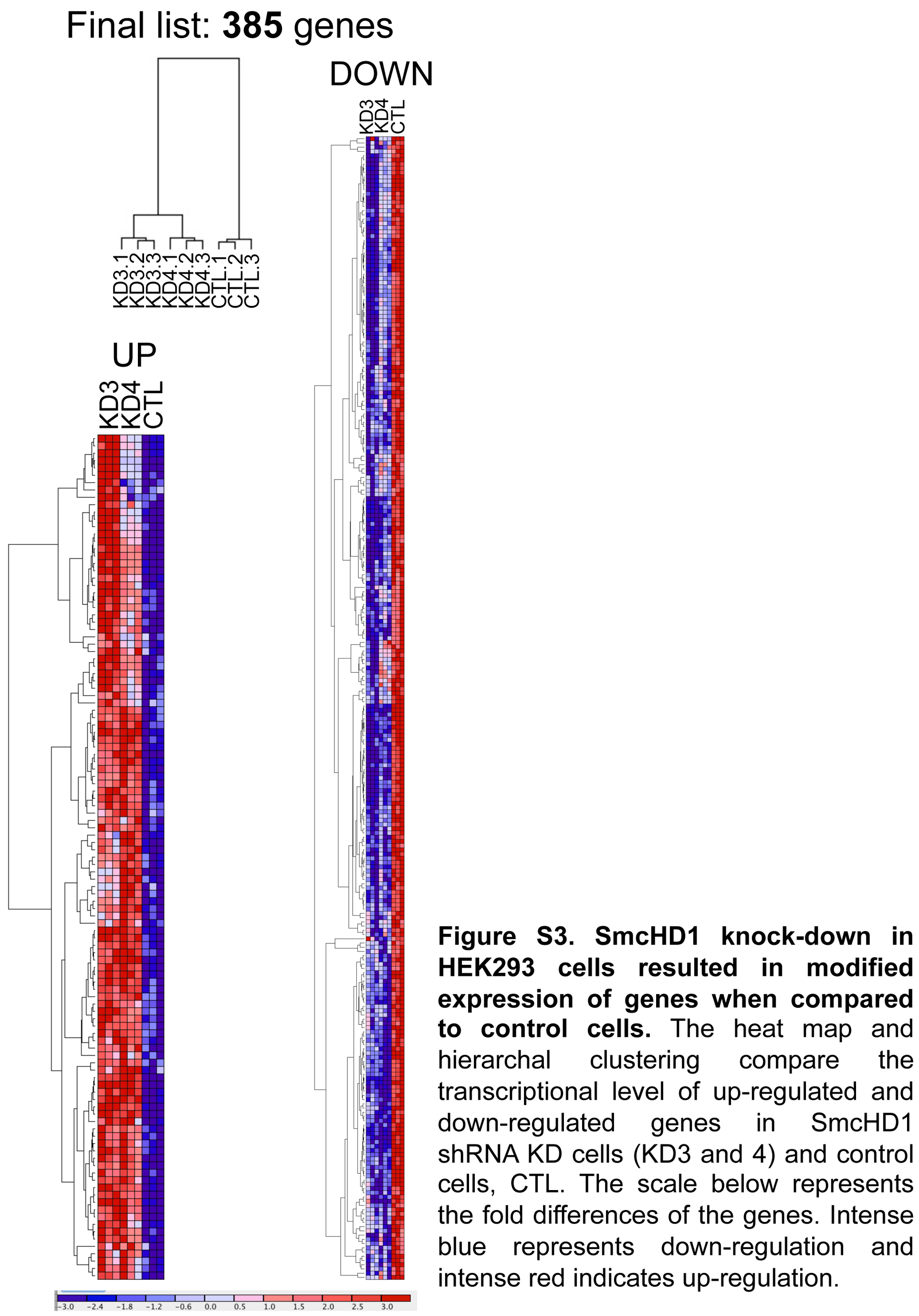

Supplement: Figure S3 — (TIF) [file pone.0097535.s003.tif]

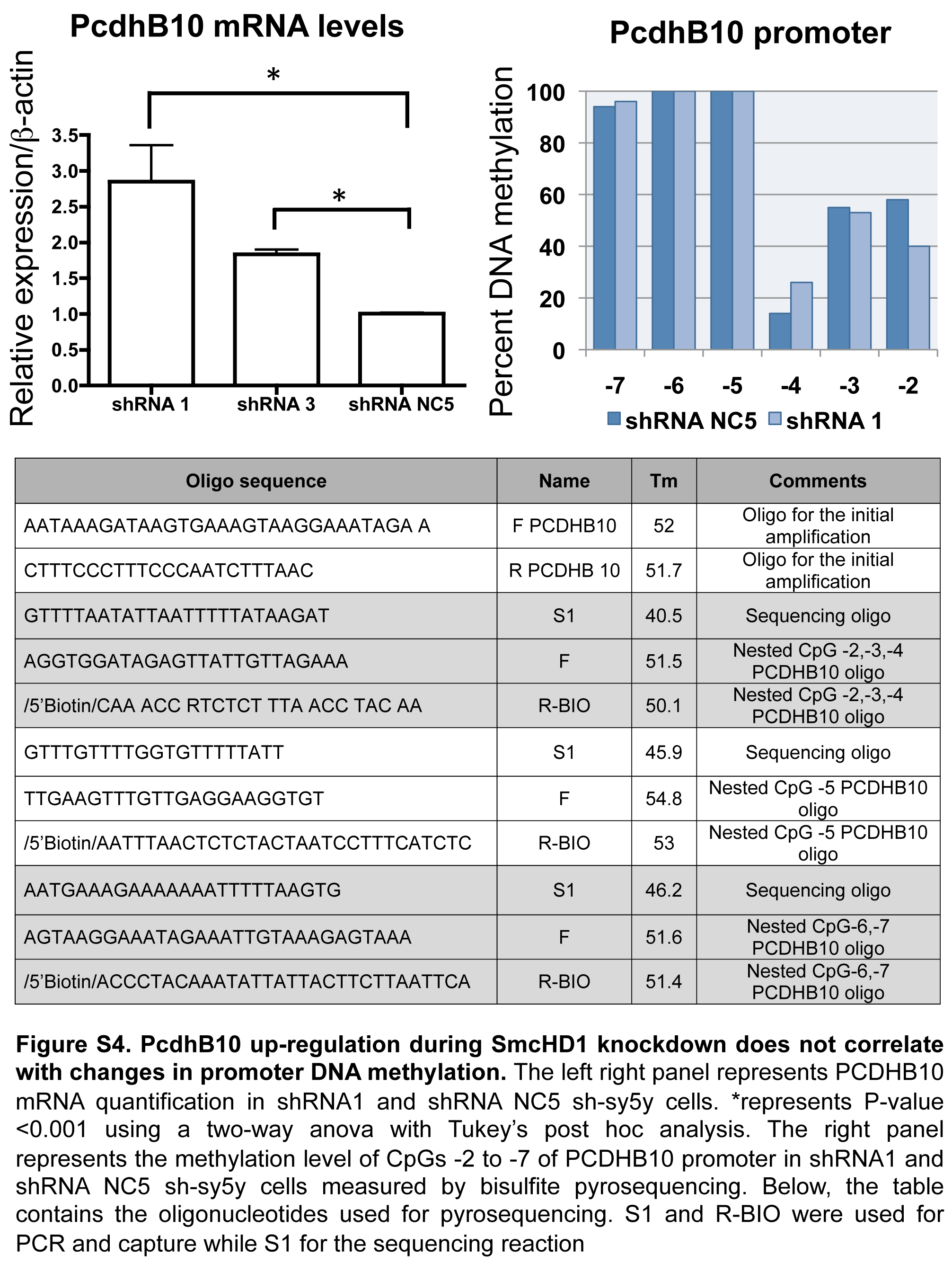

Supplement: Figure S4 — (TIF) [file pone.0097535.s004.tif]

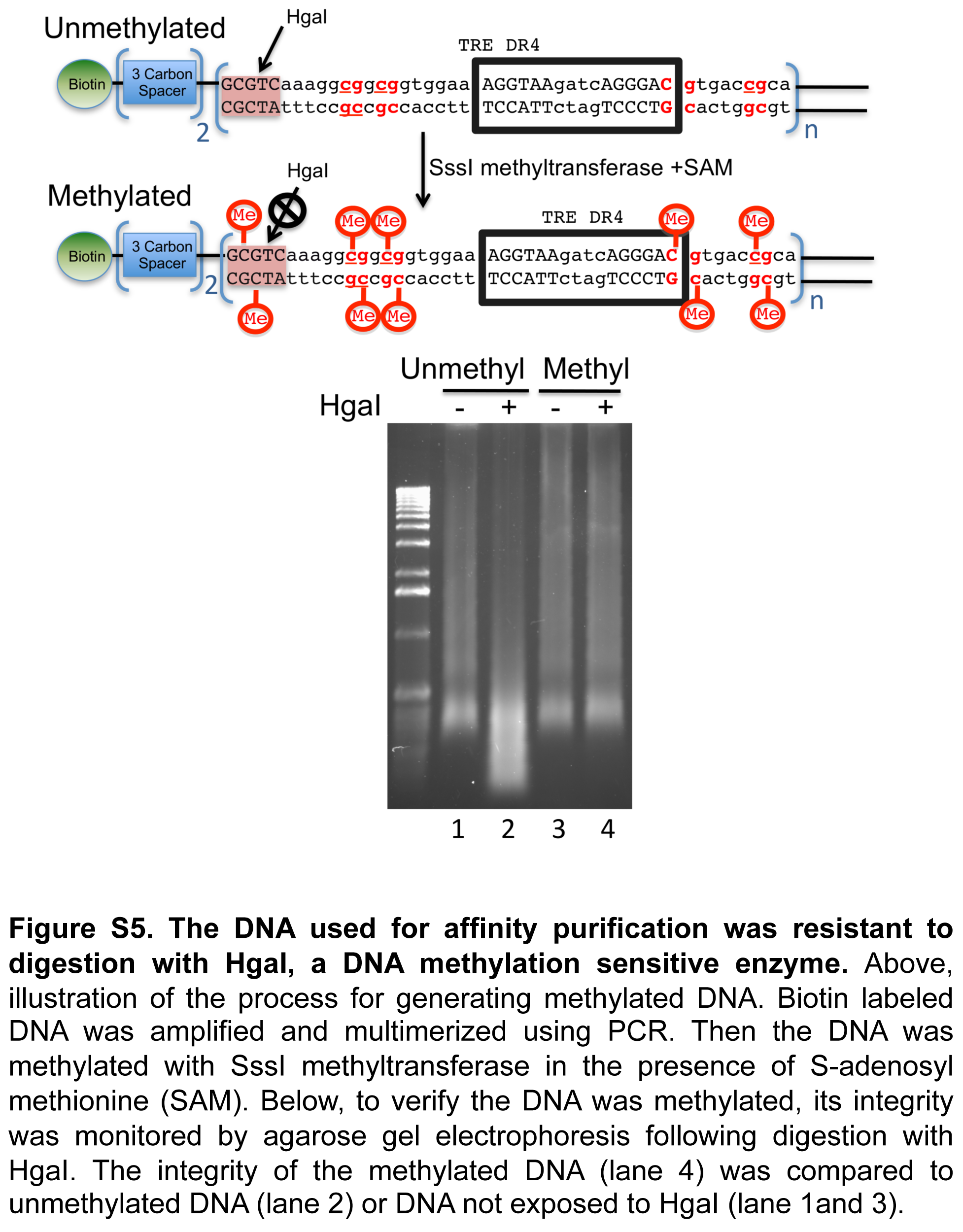

Supplement: Figure S5 — (TIF) [file pone.0097535.s005.tif]

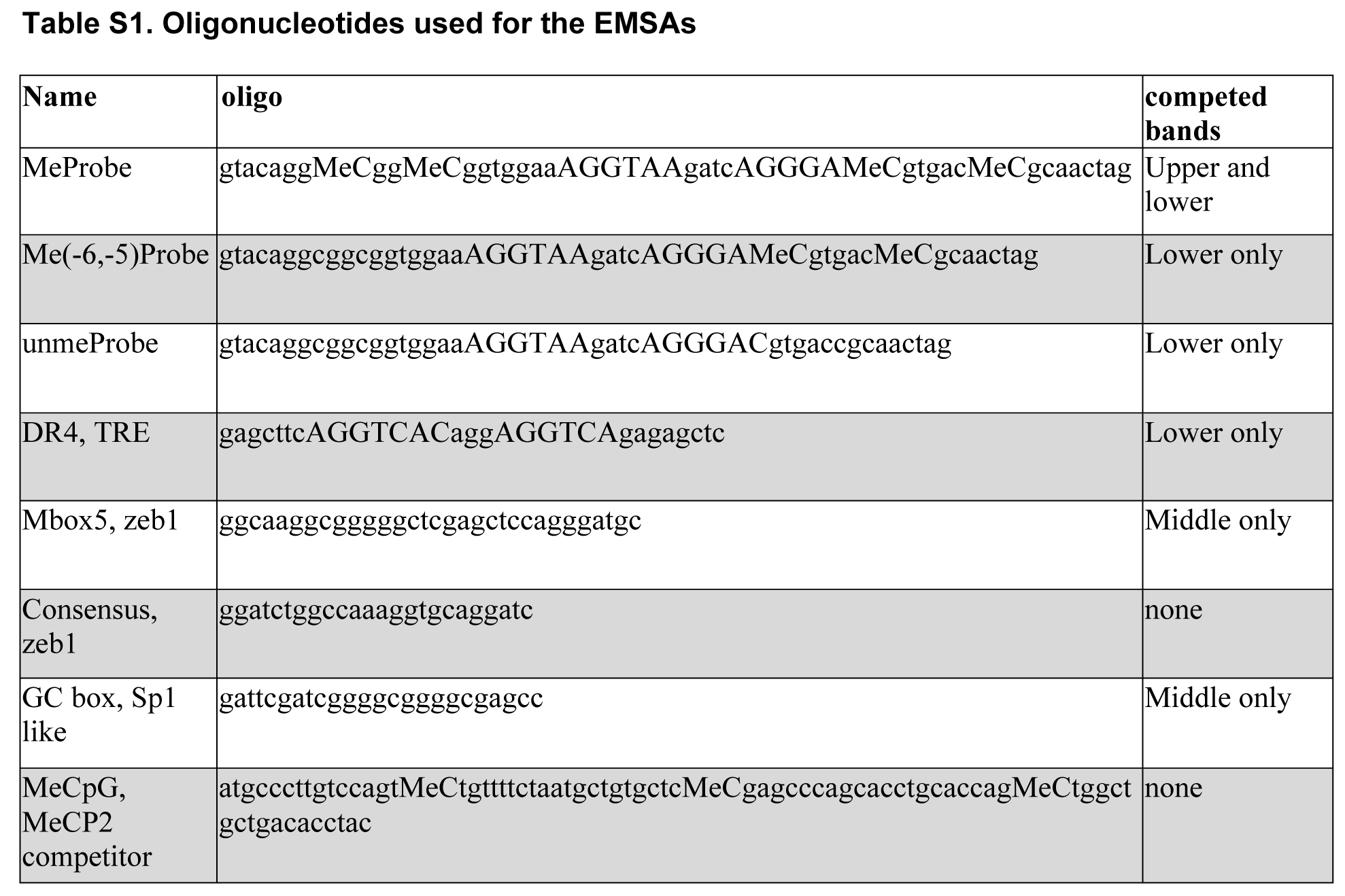

Supplement: Table S1 — (TIF) [file pone.0097535.s006.tif]

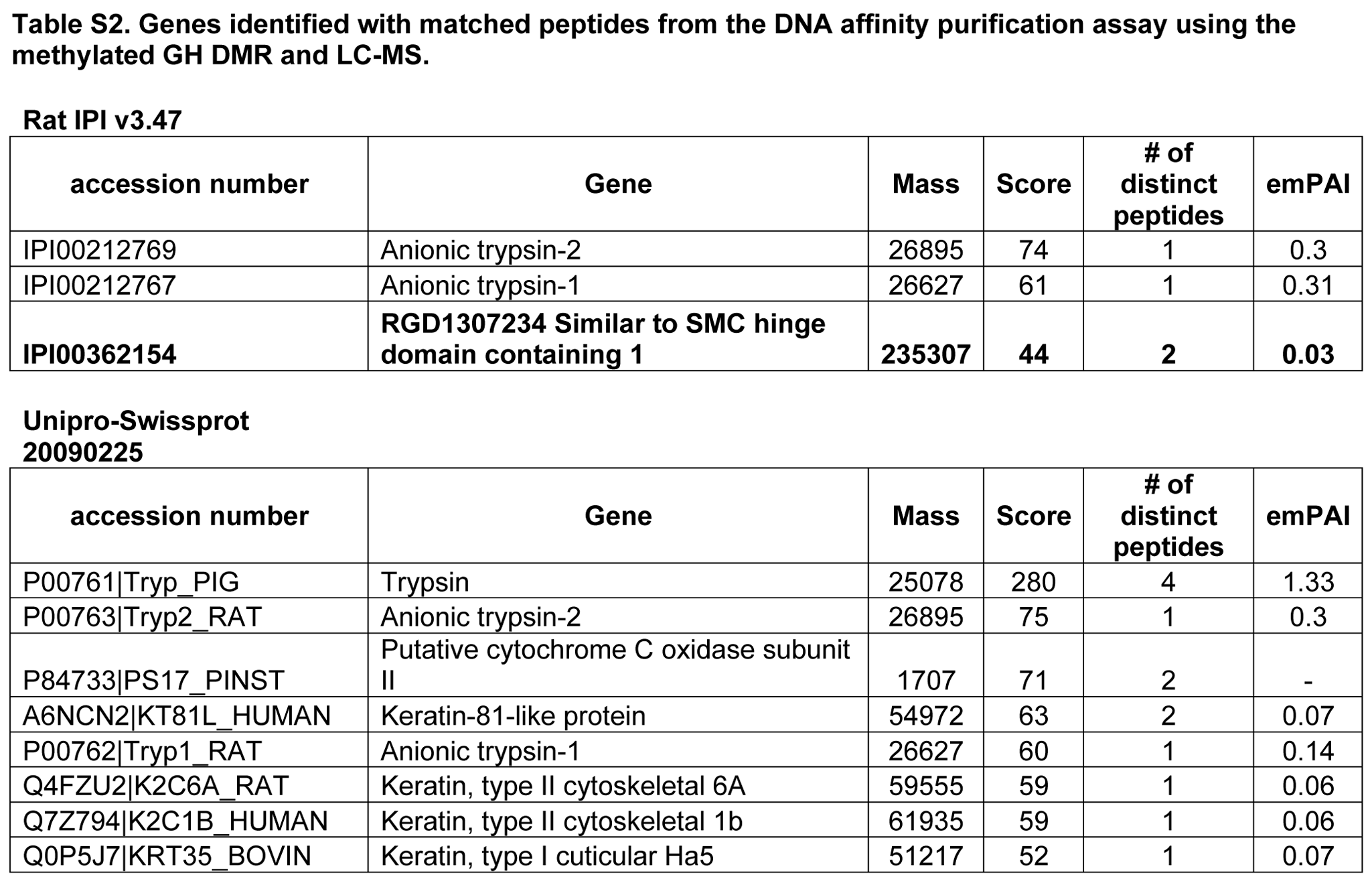

Supplement: Table S2 — (TIF) [file pone.0097535.s007.tif]

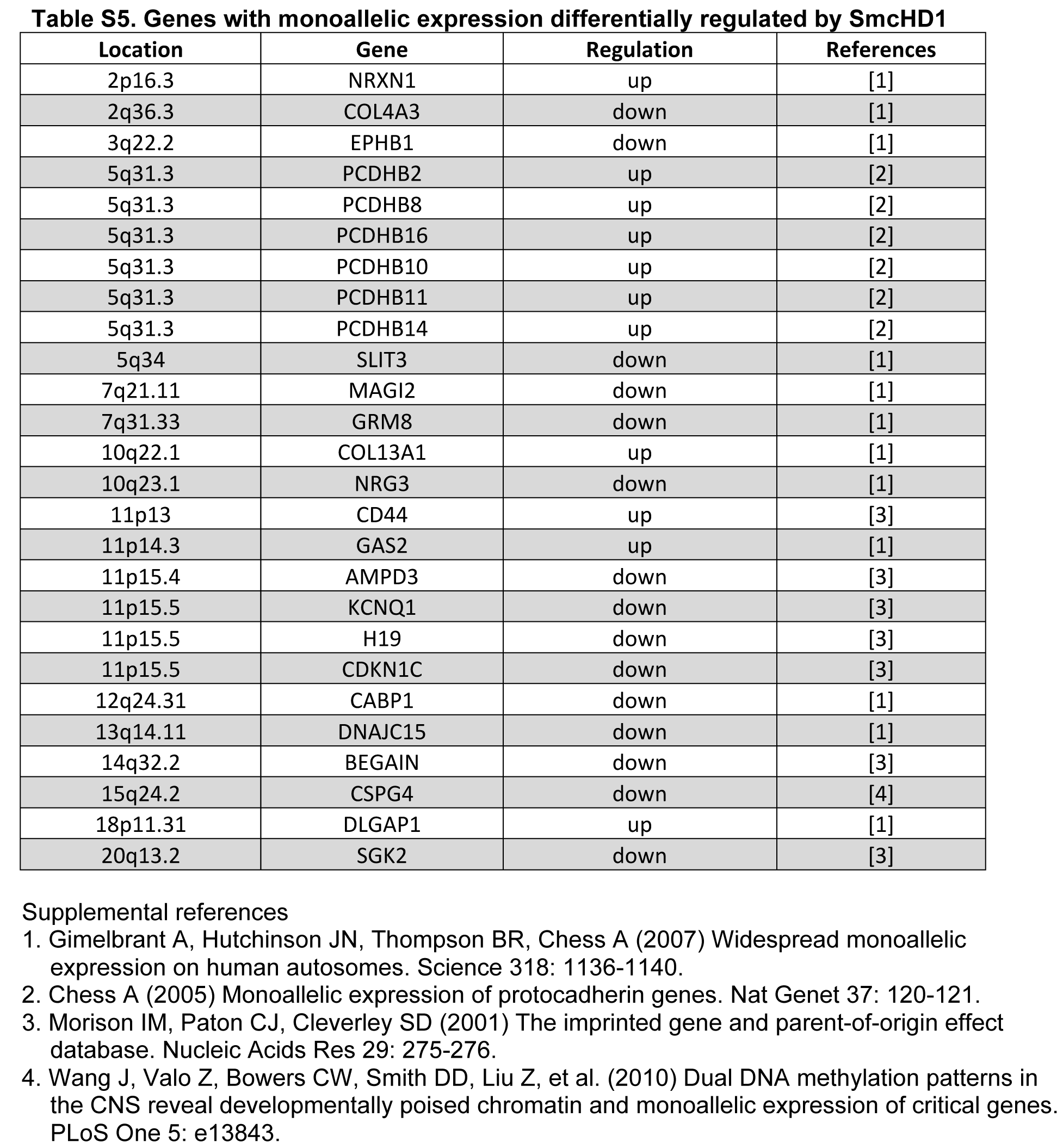

Supplement: Table S5 — (TIF) [file pone.0097535.s010.tif]

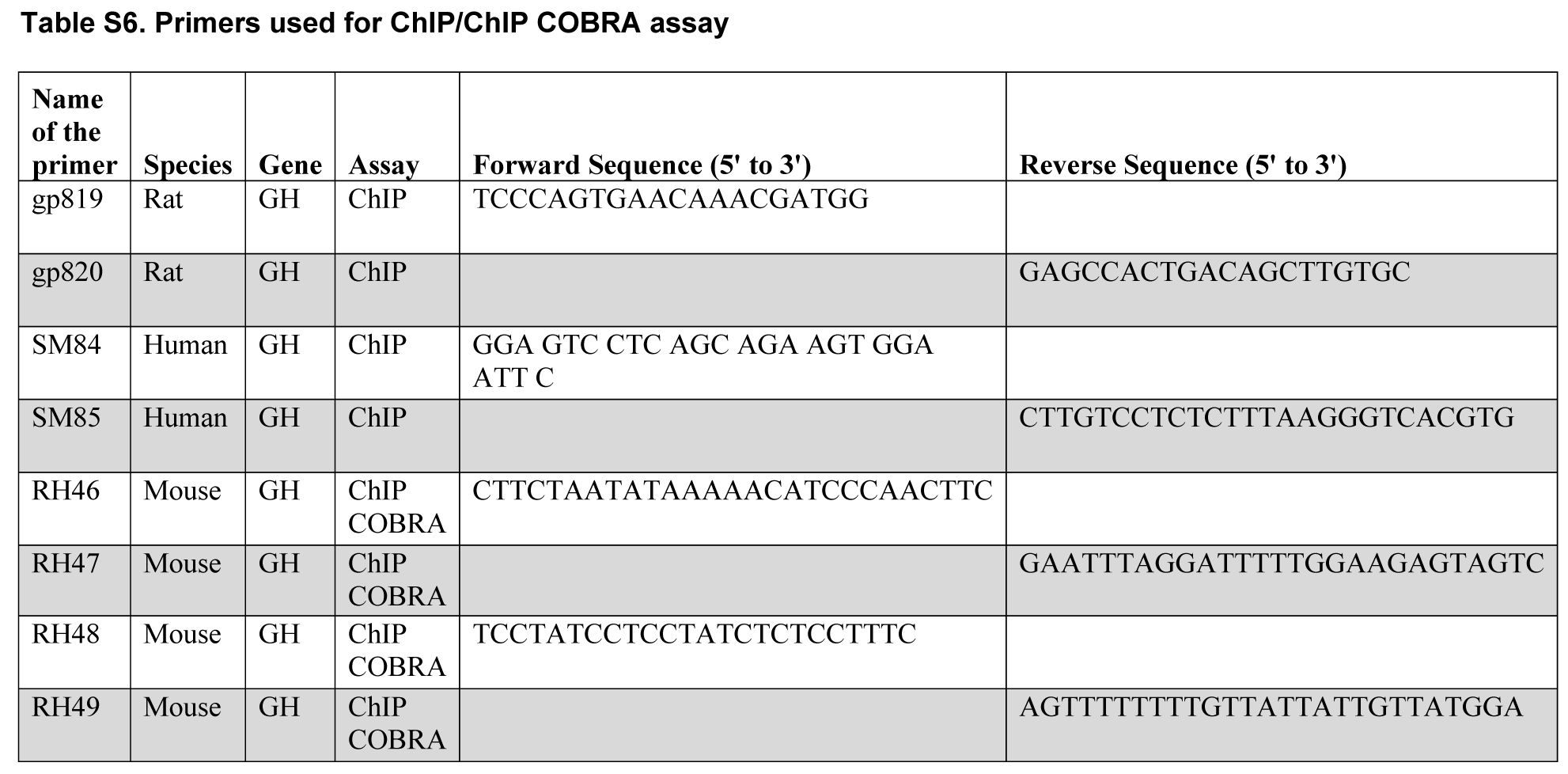

Supplement: Table S6 — (TIF) [file pone.0097535.s011.tif]

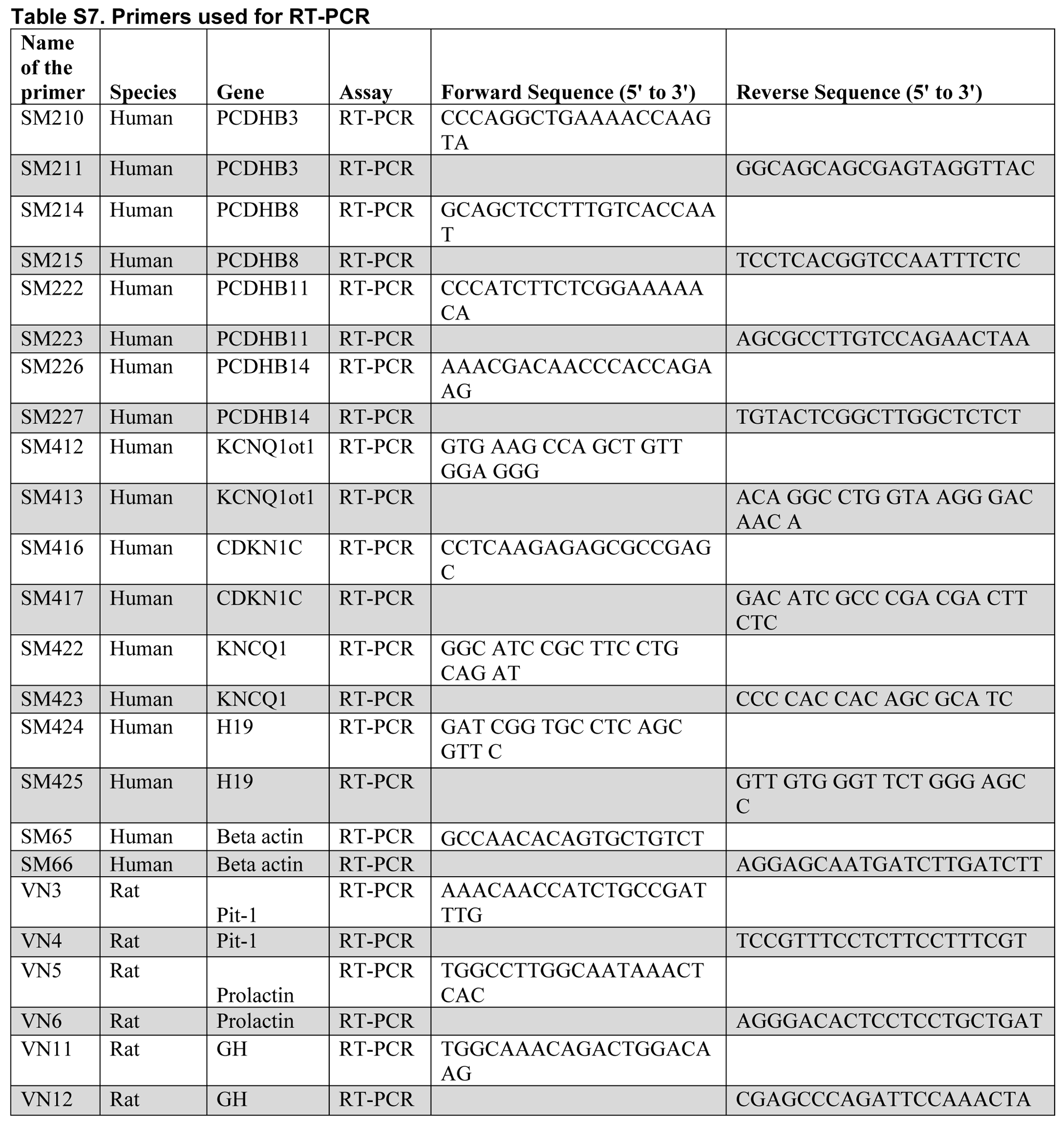

Supplement: Table S7 — (TIF) [file pone.0097535.s012.tif]

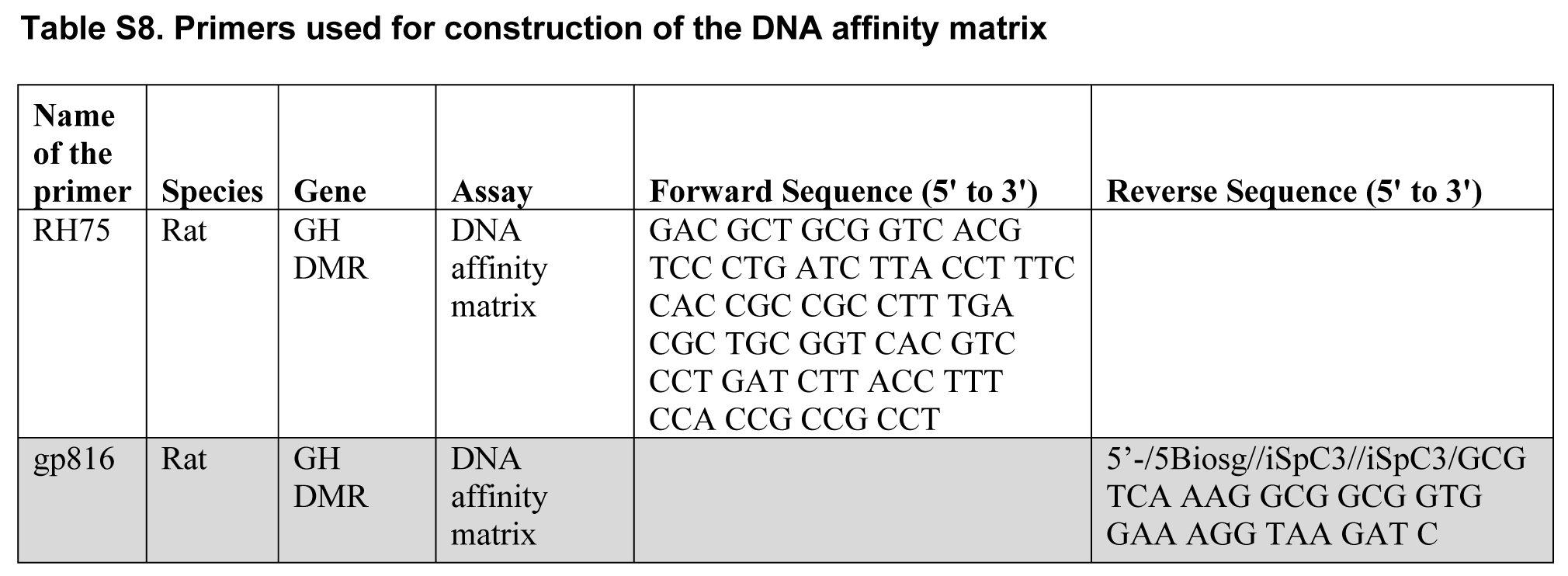

Supplement: Table S8 — (TIF) [file pone.0097535.s013.tif]

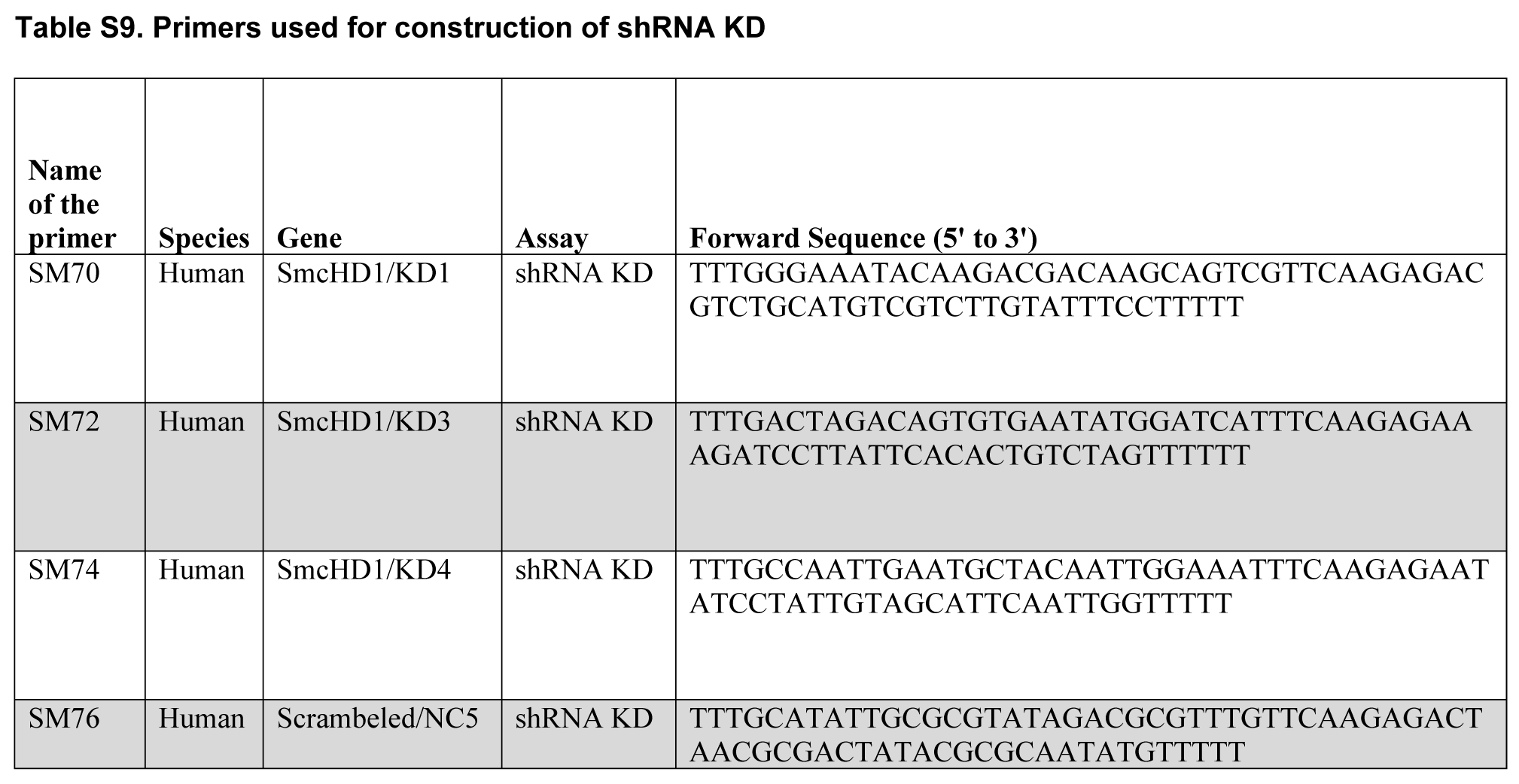

Supplement: Table S9 — (TIF) [file pone.0097535.s014.tif]
